# A Cyclin A—Myb-MuvB—Aurora B network regulates the choice between mitotic cycles and polyploid endoreplication cycles

**DOI:** 10.1371/journal.pgen.1008253

**Published:** 2019-07-10

**Authors:** Michael D. Rotelli, Robert A. Policastro, Anna M. Bolling, Andrew W. Killion, Abraham J. Weinberg, Michael J. Dixon, Gabriel E. Zentner, Claire E. Walczak, Mary A. Lilly, Brian R. Calvi

**Affiliations:** 1 Department of Biology. Indiana University, Bloomington, Indiana, United States of America; 2 Melvin and Bren Simon Cancer Center, Indianapolis, Indiana, United States of America; 3 Indiana University School of Medicine, Bloomington, Indiana, United States of America; 4 National Institute of Child Health and Human Development, National Institutes of Health, Bethesda, Maryland, United States of America; Geisel School of Medicine at Dartmouth, UNITED STATES

## Abstract

Endoreplication is a cell cycle variant that entails cell growth and periodic genome duplication without cell division, and results in large, polyploid cells. Cells switch from mitotic cycles to endoreplication cycles during development, and also in response to conditional stimuli during wound healing, regeneration, aging, and cancer. In this study, we use integrated approaches in *Drosophila* to determine how mitotic cycles are remodeled into endoreplication cycles, and how similar this remodeling is between induced and developmental endoreplicating cells (iECs and devECs). Our evidence suggests that Cyclin A / CDK directly activates the Myb-MuvB (MMB) complex to induce transcription of a battery of genes required for mitosis, and that repression of CDK activity dampens this MMB mitotic transcriptome to promote endoreplication in both iECs and devECs. iECs and devECs differed, however, in that devECs had reduced expression of E2F1-dependent genes that function in S phase, whereas repression of the MMB transcriptome in iECs was sufficient to induce endoreplication without a reduction in S phase gene expression. Among the MMB regulated genes, knockdown of AurB protein and other subunits of the chromosomal passenger complex (CPC) induced endoreplication, as did knockdown of CPC-regulated cytokinetic, but not kinetochore, proteins. Together, our results indicate that the status of a CycA—Myb-MuvB—AurB network determines the decision to commit to mitosis or switch to endoreplication in both iECs and devECs, and suggest that regulation of different steps of this network may explain the known diversity of polyploid cycle types in development and disease.

## Author summary

Endoreplication is a cell cycle variant that entails cell growth and periodic genome duplication without cell division, and results in large, polyploid cells. Cells switch from mitotic division cycles to endoreplication cycles during development, and also in response to conditional stimuli during wound healing, regeneration, aging, and cancer. Much remains unknown, however, about how mitotic cycles are remodeled into endoreplication cycles, and how similar this remodeling is between induced and developmental endoreplicating cells (iECs and devECs). In the present work, we define a Cyclin A regulated mitotic network in *Drosophila* whose downregulation promotes the switch from mitotic cycles to endoreplication cycles in both iECs and devECS. Repression of this network in iECs was sufficient to induce endoreplication without reduced expression of E2F-regulated S phase genes that is common among devECs in both flies and mice. Knockdown of downstream cytokinetic proteins, but not kinetochore proteins, were sufficient to induce different types of endoreplication. Altogether our results define a CycA—Myb—AurB network as a key determinant of alternative cell cycles, and provide insight into the regulation of a diversity of polyploid cycle types in development and disease.

## Introduction

Endoreplication is a common cell cycle variant that entails periodic genome duplication without cell division and results in large polyploid cells [1]. Two variations on endoreplication are the endocycle, a repeated G/S cycle that completely skips mitosis, and endomitosis, wherein cells enter but do not complete mitosis and / or cytokinesis before duplicating their genome again [2]. In a wide array of organisms, specific cell types switch from mitotic cycles to endoreplication cycles as part of normal tissue growth during development [1, 3]. Cells also can switch to endoreplication in response to conditional inputs, for example during wound healing, tissue regeneration, aging, and cancer [1, 4–6]. It is still not fully understood, however, how the cell cycle is remodeled when cells switch from mitotic cycles to endoreplication.

There are common themes across plants and animals for how cells switch to endoreplication during development. One common theme is that developmental signaling pathways induce endoreplication by inhibiting the mitotic cyclin dependent kinase 1 (CDK1). After CDK1 activity is repressed, repeated G / S cell cycle phases are controlled by alternating activity of the ubiquitin ligase APC/C^CDH1^ and Cyclin E / CDK2 [1]. Work in *Drosophila* has defined mechanisms by which APC/C^CDH1^ and CycE / Cdk2 regulate G / S progression, and ensure that the genome is duplicated only once per cycle [7–12]. Despite these conserved themes, how endoreplication is regulated can vary among organisms, as well as tissues within an organism. These variations include the identity of the signaling pathways that induce endoreplication, the mechanism of CDK1 inhibition, and the downstream effects on cell cycle remodeling into either an endomitotic cycle (partial mitosis) or endocycle (skip mitosis) [1, 7]. In many cases, however, the identity of the developmental signals and the molecular mechanisms of cell cycle remodeling are not known.

To gain insight into the regulation of variant polyploid cell cycles, we had previously used two-color microarrays to compare the transcriptomes of endocycling and mitotic cycling cells in *Drosophila* tissues [13]. We found that endocycling cells of larval fat body and salivary gland have dampened expression of genes that are normally induced by E2F1, a surprising result for these highly polyploid cells given that many of these genes are required for DNA synthesis. Nonetheless, subsequent studies showed that the expression of the E2F-regulated mouse orthologs of these genes is reduced in endoreplicating cells of mouse liver, megakaryocytes, and trophoblast giant cells [10, 14, 15]. *Drosophila* endocycling cells also had dampened expression of genes regulated by the Myb transcription factor, the ortholog of the human B-Myb oncogene (MYBL2) [13, 16]. Myb is part of a larger complex called Myb-MuvB (MMB), whose subunit composition and functions are mostly conserved from flies to humans [17–21]. One conserved function of the MMB is the induction of periodic transcription of genes that are required for mitosis and cytokinesis [20, 22–26]. It was these mitotic and cytokinetic genes whose expression was dampened in *Drosophila* endocycles, suggesting that this repressed MMB transcriptome may promote the switch to endocycles that skip mitosis. It is not known, however, how E2F1 and Myb activity are repressed during endocycles, nor which of the downregulated genes are key for the remodeling of mitotic cycles into endocycles.

In addition to endoreplication during development, there are a growing number of examples of cells switching to endoreplication cycles in response to conditional stresses and environmental inputs [1, 5, 6]. We will call these induced endoreplicating cells (iECs) to distinguish them from developmental endoreplicating cells (devECs). For example, iECs contribute to tissue regeneration after injury in flies, mice, humans, and the zebrafish heart, and evidence suggests that a transient switch to endoreplication contributes to genome instability in cancer [4, 6, 27–33]. Cardiovascular hypertension stress also promotes an endoreplication that increases the size and ploidy of heart muscle cells, and this hypertrophy contributes to cardiac disease [29, 34, 35]. It remains little understood how similar the cell cycles of iECs are to devECs.

Similar to the developmental repression of CDK1 activity to promote endocycles, we and others had previously shown that experimental inhibition of CDK1 activity is sufficient to induce endoreplication in flies, mouse, and human cells [28, 36–41]. These experimental iECs in *Drosophila* are similar to devECs in that they skip mitosis, have oscillating CycE / Cdk2 activity, periodically duplicate their genome during G / S cycles, and repress the apoptotic response to genotoxic stress [13, 36, 42, 43]. In this study, we use these experimental iECs to determine how the cell cycle is remodeled when cells switch from mitotic cycles to endoreplication cycles, and how similar this remodeling is between iECs and devECs. Our findings indicate that the status of a CycA—Myb—AurB network determines the choice between mitotic cycles and endoreplication cycles in both iECs and devECs.

## Results

### Induced endocycling cells have reduced expression of Myb-regulated genes

**Fig 1:**
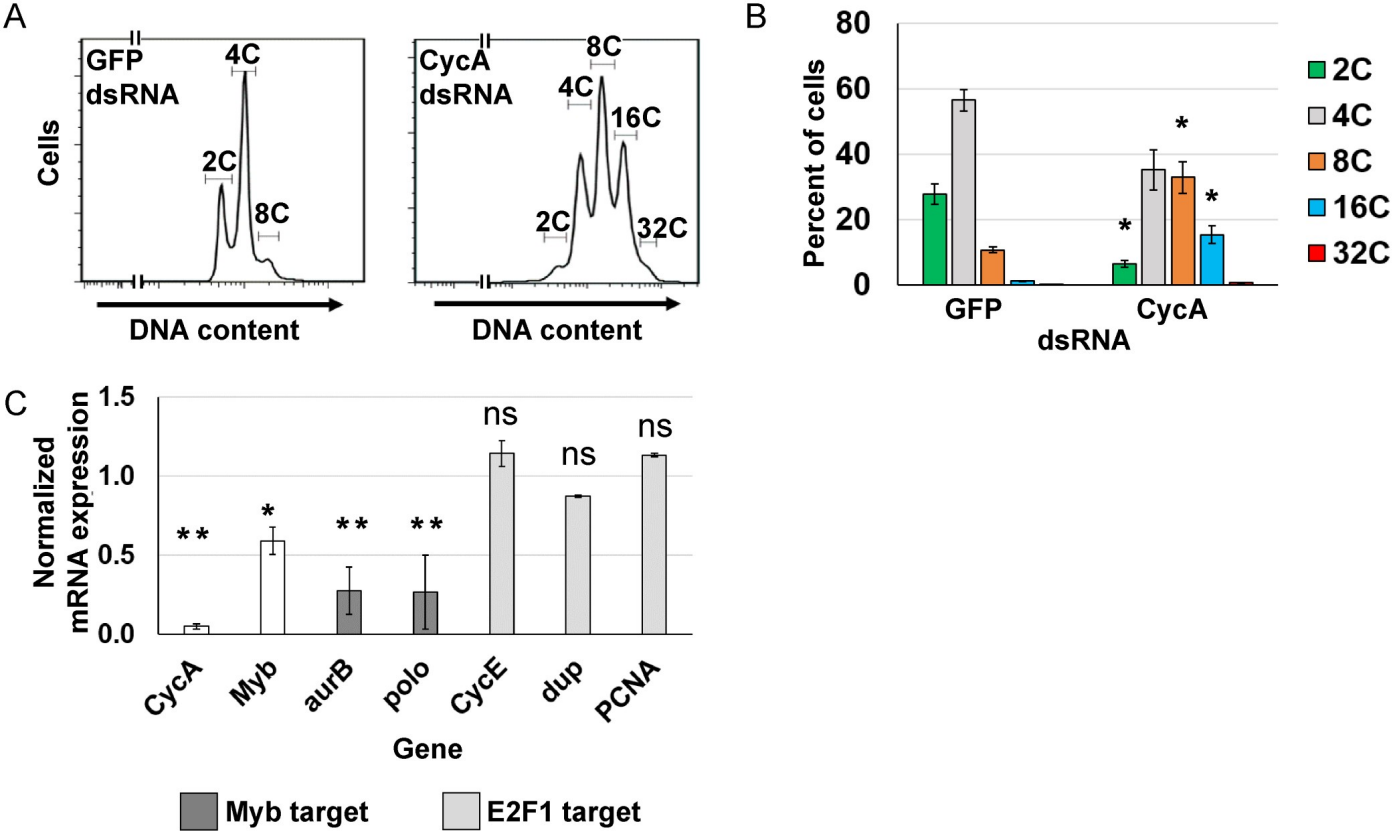
Knockdown of Cyclin A induces endoreplication cycles. **(A)** Flow cytometry of DNA content in propidium iodide labeled S2 cells 96 hours after treatment with either GFP dsRNA (control) or CycA dsRNA. **(B)** Quantification of the ploidy classes after GFP or CycA dsRNA treatment. Mean and S.E.M. for N = 3, *—p <0.05 comparing each CycA dsRNA ploidy class with the corresponding ploidy class in control GFP dsRNA treated cells. **(C)** qRT-PCR analysis of select Myb and E2F1 target gene expression in CycA dsRNA iECs. Normalized mRNA is the ratio of mRNA levels in CycA dsRNA divided by control GFP dsRNA cells (N = 3, mean and S.E.M., *—p < 0.05, **—p < 0.01, ns—not significant).

We sought to understand how remodeling of the cell cycle program determines the switch from mitotic cycles to endoreplication cycles, and how similar this remodeling is between iECs and devECs. One challenge to addressing these questions has been obtaining pure populations of cells in different cell cycles, especially for iECs that occur in tissues among a mixed population of cells. As a model for iECs, therefore, we experimentally induced *Drosophila* S2 cells in culture into endoreplication cycles by knocking down Cyclin A (CycA), which is sufficient to induce endocycles [36, 38, 44]. In *Drosophila*, CycA / CDK2 is not required for S phase, and it is believed that knockdown of CycA promotes endocycles by inhibiting CycA / CDK1 activity required for mitosis, analogous to the common mechanism of CDK1 inhibition during developmental endocycles in multiple organisms [45]. S2 cells were treated with CycA double-stranded RNA (dsRNA), and compared to a negative control population of mitotic cycling S2 cells that were treated in parallel with GFP dsRNA. Importantly, this permitted a comparison of canonical and variant cell cycles in a pure population of cells of the same cell type. Flow profiling 96 hours after treatment with CycA dsRNA indicated that more than 50% of cells had a polyploid DNA content of ≥ 8C, and a commensurate reduced fraction of cells with diploid 2C and 4C DNA contents (Fig 1A and 1B). These cells had genome doublings of 8C, 16C, and 32C that were multiples of the diploid DNA content, suggesting that they were duplicating their genomes through repeated G / S endocycles (Fig 1A and 1B). In contrast, knockdown of the mitotic Cyclin B (CycB) did not induce cells to endoreplicate, perhaps because of functional redundancy with CycB3 (S1 Fig) [46, 47]. These results confirm previous findings that inhibition of CDK activity through knockdown of CycA is sufficient to induce endoreplication in S2 cells (hereafter CycA dsRNA iEC) [36, 44].

We had previously shown that endocycling cells (G / S cycle) of the *Drosophila* larval salivary gland and fat body have dampened expression of genes that are normally induced by E2F1 and the MMB transcription factors [13]. To determine if this change in transcriptome signature also occurs in CycA dsRNA iECs, we analyzed the expression of several candidate genes whose expression is induced by E2F1 or MMB. RT-qPCR results indicated that CycA dsRNA iECs had reduced expression of the Myb subunit of the MMB and two genes that are positively regulated by the MMB and essential for mitosis (*aurora B* and *polo*) (Fig 1C). In contrast, the expression of three genes normally induced by E2F1 at G1 / S (*Cyclin E*, *PCNA*, and *dup* (fly Cdt1) were similar between CycA dsRNA iECs and mitotic cycling cells (Fig 1C). These results suggest that CycA dsRNA iECs are similar to developmental endocycling cells (devECs) in that they both have reduced expression of MMB-dependent M phase genes, but they differ in that iECs do not have reduced expression of E2F1-dependent S phase genes.

### Knockdown of *Cyclin A* or *Myb* induces similar endoreplication cycles

**Fig 2:**
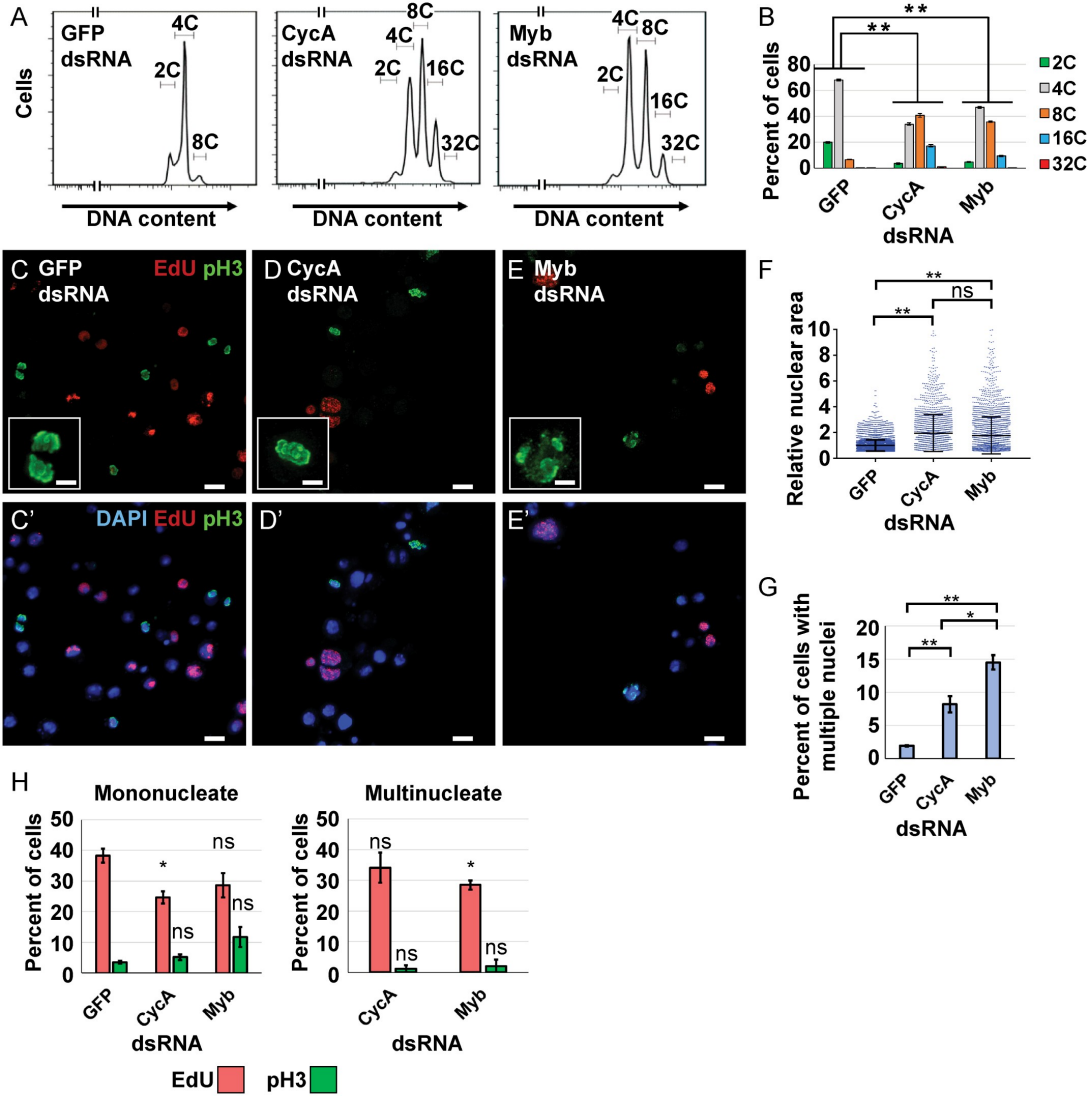
Myb or CycA knockdown induce similar endoreplication cycles. **(A)** Flow cytometry of DNA content in S2 cells treated with the indicated dsRNAs for 96 hours. **(B)** Quantification of the induced polyploidy after Myb dsRNA or CycA dsRNA treatment. Mean and S.E.M., N = 3, **—p <0.01 for each ploidy class compared to GFP dsRNA. **(C-E’)** Micrographs of cells labeled with EdU (red), pH3 (green) and DAPI (blue in **C’-E’**) after four days of treatment with dsRNA for GFP (control) **(C,C’)**, CycA **(D,D’)**, or Myb **(E,E’)**. Scale bars are 10μm. Insets are higher magnification of pH3 labelling. Scale bars are 3.3μm **(F)** Knockdown of CycA or Myb increases nuclear size. Quantification of nuclear area of S2 cells after knockdown of GFP, CycA or Myb. Each dot represents the nuclear area of a single cell divided by the mean area of GFP controls (machine units) (Mean and S.D. N = 3, *—p < 0.05, **—p < 0.01, ns—not significant). **(G)** Quantification of the fraction of total S2 cells with multiple nuclei after the indicated treatment (mean and S.E.M. for N = 3, *—p<0.05, **—p<0.01 relative to GFP dsRNA). **(H)** Quantification of EdU and pH3 labeling in mononucleate and multinucleate cells after treatment with the indicated dsRNAs (mean and S.E.M. for N = 3, *—p < 0.05, ns—not significant).

Although CycA dsRNA iECs had lower expression of two MMB-induced genes that are required for mitosis, it was unclear whether dampened MMB activity contributed to the switch to endoreplication. To address this question, we knocked down expression of the Myb subunit of the MMB, which is required to induce the expression of genes for mitosis and cytokinesis [22–24]. Knockdown of Myb inhibited cell proliferation, and resulted in an increase in polyploid DNA content that was similar to that of CycA dsRNA iECs (Fig 2A and 2B, S2 Fig). We then used fluorescence microscopy to further evaluate ploidy and cell cycle in CycA and Myb knockdown cells. S phase cells were detected by incubating in the nucleotide analog EdU for two hours followed by fluorescent click-it labeling, M phase cells detected with antibodies against phospho-histone H3 (pH3), and nuclear DNA labeled with DAPI [48–50]. Treatment of cells with either CycA or Myb dsRNA resulted in a similar frequency and size of large polyploid nuclei, indicating that Myb knockdown induced endoreplication (hereafter Myb dsRNA iEC) (Fig 2C–2F). There was a higher fraction of multinucleate Myb dsRNA iECs (~15%) than CycA dsRNA iECs (~8%), suggesting that Myb knockdown results in a somewhat larger fraction of endomitotic cells than does CycA knockdown (Fig 2G). Approximately 30% of CycA dsRNA iECs and Myb dsRNA iECs incorporated EdU, an S phase fraction that was similar in both mononucleate and multinucleate populations, consistent with periodic duplications of the genome during both endocycles and endomitotic cycles (Fig 2H). Despite this evidence for periodic endoreplication, the fraction of total cells with mitotic PH3 labeling was not decreased after CycA knockdown (~5%), and was slightly increased after Myb knockdown in the mononucleate population (~10%) (Fig 2H). Unlike control mitotic cells, however, the PH3 labeling after CycA and Myb knockdown was diffuse, with little evidence of fully condensed mitotic chromosomes, suggesting that these cells were either arrested or delayed in early mitosis or endomitosis, and are consistent with previous observations of chromosome condensation defects of Myb mutants *in vivo* [51] (Fig 2C–2E insets). These results indicate that knockdown of Myb is sufficient to induce endoreplication cycles that are similar to those after knockdown of CycA.

### Myb induction of M phase gene expression is dependent on CycA

**Fig 3:**
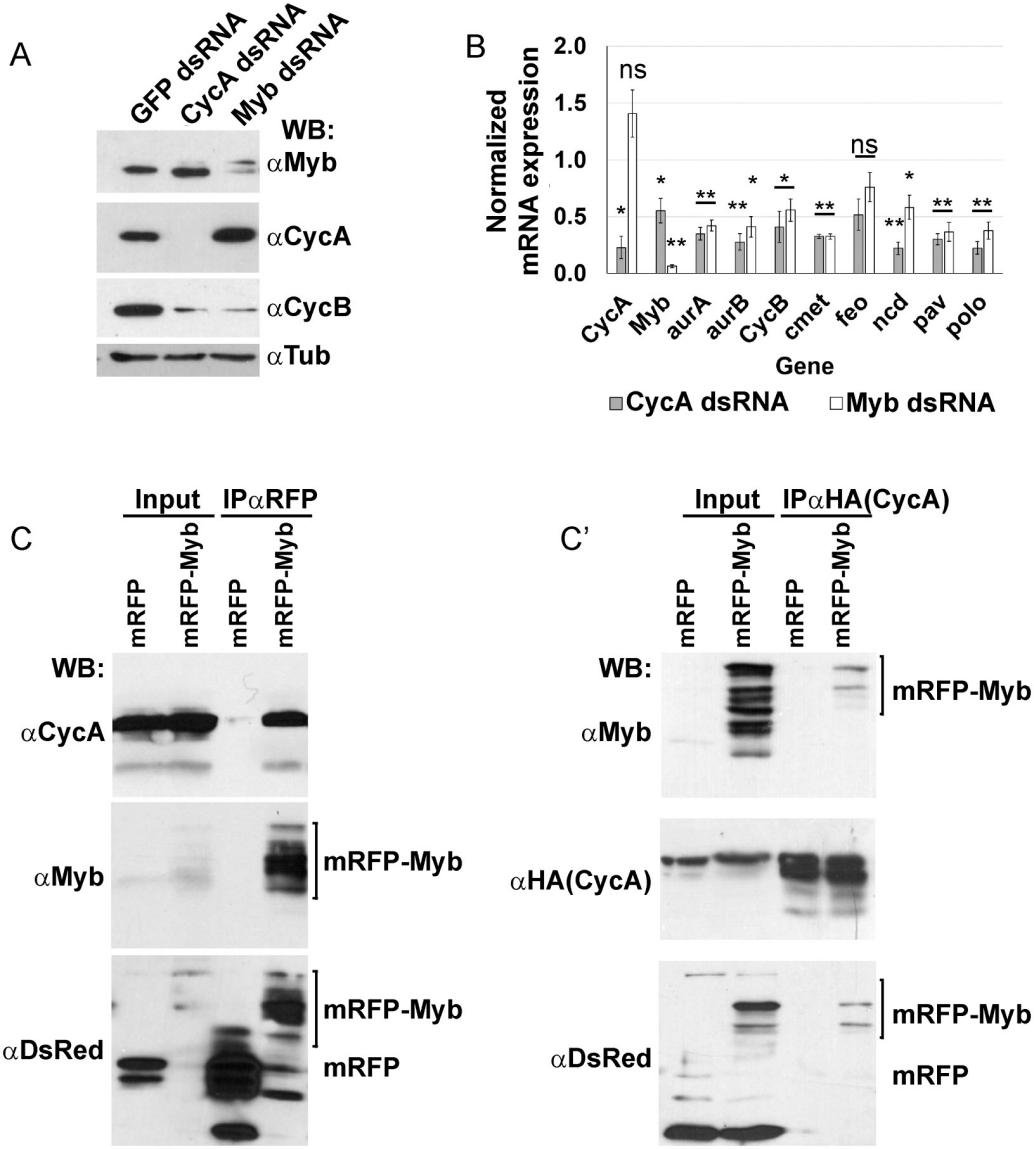
Myb induction of M phase gene expression is downstream of and dependent on CycA. **(A)** Cyclin B protein levels are reduced in CycA^dsRNA^ and Myb^dsRNA^ iECs. Western blot of S2 cell extracts after treatment with the indicated dsRNA and incubated with antibodies against CycA, Myb, CycB, and alpha-Tubulin (loading control) (N = 3, a representative blot is shown). **(B)** RT-qPCR analysis of select MMB target gene expression in CycA^dsRNA^ and Myb^dsRNA^ iECs. Values shown are the mRNA levels in knockdown cells divided by those in control GFP^dsRNA^ cells (mean and S.E.M. for N = 3 biological replicates, *—p < 0.05, **—p < 0.01, ns—not significant). **(C)** CycA and Myb proteins interact *in vivo*. Larvae expressing *UAS-CycA* and either *UAS-mRFP* or *UAS-mRFP-Myb* were immunoprecipitated with nanobodies against mRFP and then Western blotted (WB) with antibodies against CycA, Myb, and DsRed (detects mRFP) (representative blot, N = 3), **(C’)** Reciprocal IP. Larvae expressing *UAS-CycA-HA* and either *UAS-mRFP* or *UAS-mRFP-Myb* were immunoprecipitated with antibodies against HA and then Western blotted (WB) with antibodies against Myb, HA to detect CycA:HA, and DsRed (representative blot, N = 3).

The similarity between CycA dsRNA and Myb dsRNA iECs suggested that CycA and Myb may have a functional relationship. It had been shown in human cells that CycA / CDK2 phosphorylates Myb and promotes its activity as transcription factor [52, 53]. These early studies, however, were before the discovery that Myb acts as part of the MMB and the identification of many MMB regulated genes [54, 55]. Moreover, it is not known whether CycA regulation of Myb is conserved in *Drosophila*. To begin to address this question, we analyzed iECs by Western blotting. The results showed that CycA and Myb dsRNA treatments resulted in the expected lower levels of their respective proteins (Fig 3A). Importantly, both CycA and Myb dsRNA iECs also had greatly reduced levels of CycB protein, consistent with the known requirement of the MMB for transcriptional induction of *CycB* during mitotic cycles, and further suggesting that CycA knockdown may compromise MMB activity (Fig 3A) [24, 29, 56]. To further address this possibility, we used RT-qPCR to quantify mRNA levels for CycB and other known MMB target genes that function in mitosis or cytokinesis. Knockdown of either CycA or Myb reduced the expression of all these MMB target genes to similar extents (Fig 3B). Knockdown of CycA resulted in reduced Myb mRNA, even though the Western results showed that there was no reduction of Myb protein. This result is consistent with previous reports that the periodic proteolysis of Myb, which normally occurs during mitosis, is absent during endoreplication cycles [57]. In contrast, knockdown of Myb did not reduce levels of either CycA mRNA or protein, suggesting that Myb knockdown is sufficient to induce endoreplication cycles even when CycA protein levels are high (Fig 3A and 3B). These results suggest that CycA complexed with either CDK1 or CDK2, is required for MMB transcriptional activation of M phase genes.

To further evaluate CycA regulation of the MMB, we determined if Myb and CycA physically interact. We used the GAL4 / UAS system to express *UAS-CycA* with either *UAS-Myb-RFP* or *UAS-RFP* in mitotic cycling imaginal discs, immunoprecipitated (IPed) Myb-RFP or RFP with an anti-RFP nanobody, and then blotted for Cyclin A [25, 58]. The results indicated that Myb-RFP, but not RFP alone, co-IPs with CycA (Fig 3C). The IP’ed RFP-Myb protein reproducibly migrated as a cluster of four bands, which could be the result of post-translational modification, although lower molecular weight species specifically recognized by an anti-dsRed antibody suggests some proteolysis had occurred (Fig 3C). In the reciprocal experiment, IP of CycA-HA with HA antibodies co-IPed Myb-RFP but not RFP alone (Fig 3C’). All together, these results are consistent with the hypothesis that during *Drosophila* mitotic cycles a CycA / CDK complex is directly required for the MMB to induce expression of genes required for M phase, and that in the absence of this activation cells switch to endoreplication cycles.

### iECs have reduced expression of Myb-regulated genes that function at multiple steps of mitosis and cytokinesis

To further evaluate the relationship between CycA and Myb and gain insight into remodeling of mitotic cycles into endoreplication cycles, we analyzed the global transcriptomes of CycA dsRNA and Myb dsRNA iECs by RNA-Seq. The transcriptome of these two iEC populations were compared to control mitotic cycling S2 cells treated in parallel with GFP dsRNA, all in three biological replicates. Genes were defined as differentially expressed (DE) in iECs if their normalized steady state mRNA levels differed from mitotic cycling cells with a log2 fold change (log2FC) of at least +/- 0.5 and a false discovery rate (FDR) corrected p-value <0.05 [59].

**Fig 4:**
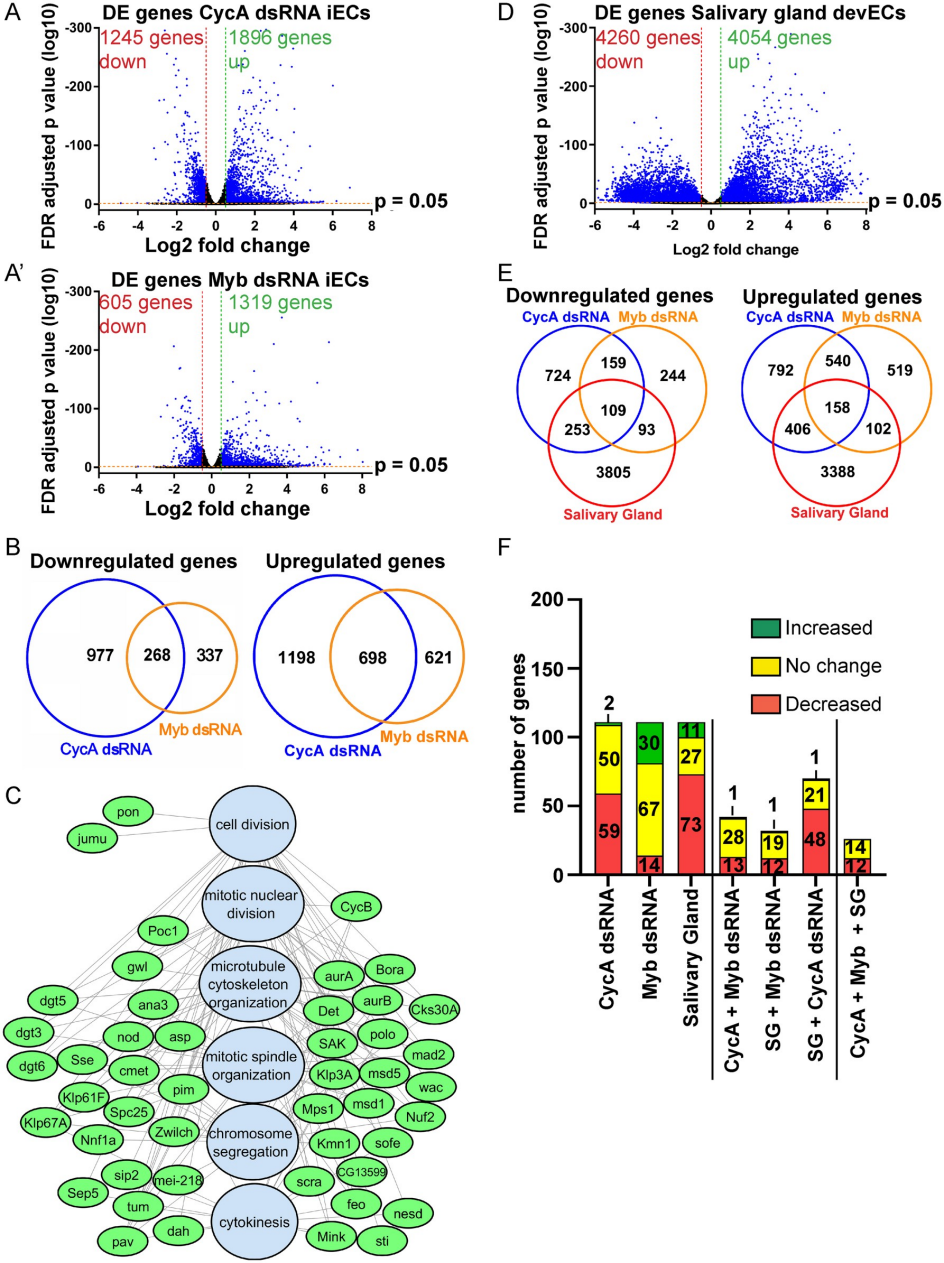
Both iECs and devECs have reduced expression of Myb target genes that function at multiple steps of mitosis and cytokinesis. **(A, A’)** Volcano plots of RNA-Seq results for differentially expressed (DE) genes in CycA dsRNA iECs **(A)** and Myb dsRNA iECs **(A’)** each relative to GFP dsRNA control cells (N = 3 biological replicates). Vertical red and green dotted lines indicate thresholds for log2 fold change (≤ -0.5 and ≥+0.5) in iECs and horizontal red line the FDR adjusted p-value = 0.05. Blue dots represent genes that fulfill both of these criteria. See also S1 and S2 Tables. **(B)** Venn diagrams comparing the overlap of DE genes in CycA dsRNA and Myb dsRNA relative to control GFP dsRNA cells. See also S3 Table. **(C)** Gene Ontology (GO) analysis of genes downregulated in iECs and devECs indicate an enrichment for Myb target genes that are required for mitosis. Shown is a network analysis with GO biological process categories in blue and downregulated genes in green. See also S3A Fig and S4 Table. **(D)** Volcano plot of RNA-Seq results for DE genes in endocycling cells from early 3^rd^ instar larval salivary glands relative to mitotic cycling larval brains and imaginal discs from the same animals (N = 3 biological replicates). Vertical red and green dotted lines indicate thresholds for log2 fold change (≤ -0.5 and ≥+0.5) and horizontal red line the FDR adjusted p-value = 0.05. Blue dots represent genes that fulfill both of these criteria. See also S4 and S5 Tables. **(E)** Venn diagrams showing the overlap of DE genes in iECs with DE genes in salivary gland (SG) devECs (the latter relative to mitotic brains and discs). See also S3B Fig and S5 Table. (**F**) Meta-analysis of 111 E2F1-dependent genes (Dimova categories A+B+C) [61] in iECs and SG devECs. The first three bars represent the number of E2F1-dependent genes that were increased, decreased, or unchanged relative to mitotic cycling controls for each endoreplicating cell type (total 111 E2F1-dependent genes). The other bars represent E2F1-dependent genes whose expression relative to mitotic cycling cells was similar among pairs or all three endoreplicating cell types. See also S6 Table.

The RNA-Seq results indicated that a switch from mitotic cycles to endoreplication in CycA dsRNA and Myb dsRNA iECs is associated with differential expression of thousands of genes (Fig 4A and 4A’, S1 and S2 Tables). Comparison of the CycA dsRNA and Myb dsRNA iEC transcriptomes revealed that they shared a total of 966 genes that were differentially expressed compared to mitotic cycling controls (698 increased and 268 decreased) (Fig 4B, S3 Table). Permutation testing indicated that this overlap of DE genes was highly statistically significant, with the overlap in upregulated genes being 4.6 fold greater than expected by chance (p<1 x 10^−5^), and that of downregulated genes being 5.8 fold greater than expected by chance (p<1 x 10^−5^) (S3A Fig). Analysis of Gene Ontology (GO) biological process categories indicated that the upregulated genes shared by CycA dsRNA iEC and Myb dsRNA iECs were significantly enriched in the categories of immunity, metabolism, and development (q < 5 x 10^−4^), and that shared down regulated genes also included those for energy metabolism (q ≤ 1 x 10^−9^) (S4 and S5 Figs, S3 Table) [60].

With respect to cell cycle remodeling, the downregulated genes shared by these two iEC types were significantly enriched for multiple GO categories of mitosis and cytokinesis (q < 1 x 10^−9^) (Fig 4C, S5 Fig, S4 Table). After removing redundant GO categories, we analyzed the genes from the five most significantly enriched categories. These categories comprise 47 genes with functions in mitosis and cytokinesis that were downregulated by up to several fold in both iEC types (Fig 4C, S4 Table). Georlette and colleagues had previously shown that many of these genes require the Myb subunit of the MMB for their expression in *Drosophila* Kc cells [24]. The common downregulation of these genes in CycA dsRNA iEC and Myb dsRNA iEC further suggests that CycA is required for the MMB to induce transcription of these mitotic genes, and that downregulation of a subset of the MMB transcriptome in these two iEC types may contribute to the switch from mitotic cycles to endoreplication cycles.

### The MMB mitotic transcriptome is downregulated in both iECs and devECs

The RNA-seq results, together with our published analysis of devECs, suggested that iECs are similar to devECs in that both have a dampened Myb transcriptome of mitotic genes [13]. However, our previous analysis of devECs used two-color microarrays that had a limited gene set and sensitivity [13]. Therefore, to more fully compare iEC and devEC transcriptomes, we expanded the analysis of devECs with RNA-Seq. Specifically, we used RNA-Seq to compare the transcriptome of endocycling larval salivary glands (SG) to that of mitotic cycling larval brains and discs (B-D) from early third instar larvae, all in three biological replicates. The results indicated that 4,054 genes were upregulated and 4,260 genes downregulated in SG devECs relative to mitotic cycling B-D cells (log2FC at least +/- 0.5 and corrected p-value <0.05) (Fig 4D).

A comparison of SG devEC with CycA dsRNA and Myb dsRNA iECs showed that they had in common 158 genes that are increased and 109 genes that are decreased in expression relative to mitotic cycling cells (Fig 4E, S5 Table). This observed overlap in upregulated genes among all three endoreplicating cell types was 4.3 fold greater than expected by chance (p<1 x 10^−5^), while the overlap of downregulated genes was 9.1 fold greater than expected by chance (p<1 x 10^−5^) (S3B Fig). Consistent with our previous array analysis, many genes induced by E2F1 and MMB are expressed at lower levels in endocycling SG devECs relative to mitotic cycling B-D cells [13]. Among the 111 genes that are known to depend on E2F1 for their transcriptional induction in S2 cells, 73 were reduced in expression in SG devEC (Fig 4F, S6 Table) [61, 62]. Fewer E2F1-dependent genes (59) were downregulated in CycA dsRNA iECs, with an overlap of 48 downregulated E2F1-dependent genes in both CycA dsRNA iECs and SG devECs (Fig 4F, S5 Table) [61]. 11 of the 25 E2F1-dependent genes that were downregulated in devECs but not in CycA dsRNA iECs have functions in S phase, including *CycE*, *dup* (Cdt1), and *PCNA*; the three E2F1-regulated genes that RT-qPCR had indicated are not repressed in CycA dsRNA iECs (Fig 1C, S5 Table). Thus, although reduced expression of these E2F1-regulated S phase genes is common in devECs, their repression is not essential for endoreplication [10, 13–15]. Consistent with this idea, only 12 E2F1-dependent genes were commonly downregulated in both iEC types and devECs, and all have functions in mitosis (Fig 4F, Table 1, S6 Table). These 12 E2F1-dependent genes are a subset of the 47 Myb-dependent mitotic genes that we had found are downregulated in iEC, and therefore require both E2F1 and the MMB for their full expression (Fig 4C, S6 Table) [62].

**Table 1:** Downregulated E2F1 and Myb dependent genes common to iECs and devECs.

Gene abbreviation	Function
tum	Regulation of cytokinesis
pav	Regulation of cytokinesis
sti	Regulation of cytokinesis
Nnf1a	Component of kinetochore
Kmn1	Component of kinetochore
Klp67A	Regulation of mitotic spindle assembly
mad2	Component of Spindle Assembly checkpoint
polo	Spindle assembly, Spindle assembly checkpoint, cytokinesis
msd1	Facilitates microtubule nucleation for chromosome segregation
msd5	Centrosome independent nucleation of microtubules
nod	meiotic chromosome segregation
CG7341	Unknown

Considering all downregulated genes, the most significantly enriched GO categories shared by iECs and devECs were mitosis and cytokinesis, including all of the 47 Myb-dependent genes that were commonly downregulated between CycA and Myb dsRNA iECs (Fig 4C, S6 Fig, S3 and S4 Tables). Given that CycA / Cdk1 activity is repressed in both CycA dsRNA iEC and SG devECs, the lower expression of these 47 genes in devECs further suggests that their transcriptional induction by the MMB is dependent on CycA (S4 Table) [9, 11, 63]. These genomic results show that while iECs and devECs both have a dampened MMB transcriptome of mitotic genes, repression of E2F1-regulated S phase genes is not essential for endoreplication.

### Integration of genetic analysis with RNA-Seq implicates a CycA—Myb—AurB network in endoreplication control

**Fig 5:**
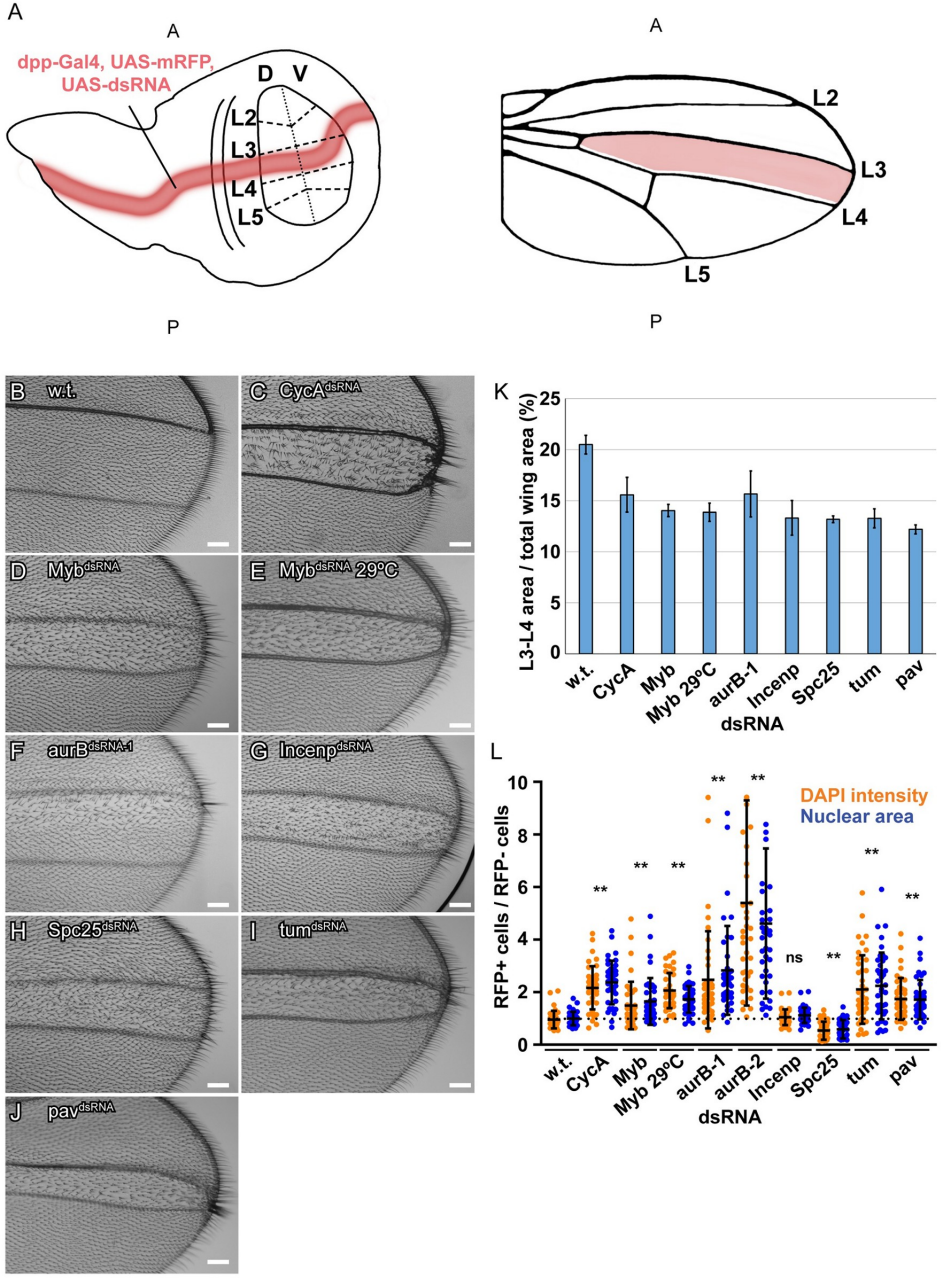
Integration of a genetic screen with RNA-Seq implicates a CycA-Myb-AurB network in endoreplication control. (**A**) Screen strategy: *dpp-GAL4* was used to express *UAS-mRFP* and *UAS-dsRNAs* along the A-P compartment boundary of wing discs (red stripe in wing disc on left). These cells are fated to form the central part of the adult wing (red stripe in wing on right). This central wing region was screened for larger more widely spaced hairs. (**B-J**) Results from an RNAi screen of candidate genes that were expressed at lower levels in iECs. Shown are brightfield images of an adult wing from animals that expressed *dpp-GAL4* and the indicated *UAS-dsRNA* along the A-P boundary of larval wing discs. **(B)** A control wild type (w.t.) wing from a *dpp*:*GAL4*; *UAS-GFP* animal. **(C)** A wing from a *dpp-GAL4; UAS-CycA*^*dsRNA*^ animal. Note clusters of larger and thicker hairs on the wing surface along the A-P boundary as well as on the distal wing margin between veins L3 and L4. **(D-I)** Adult wings after expression of *UAS-Myb*^*dsRNA*^ **(D)**, *UAS-Myb*^*dsRNA*^ at 29 C (E), *aurB*^*dsRNA-1*^ **(F)**, *Incenp*^*dsRNA*^ **(G)**, *Spc25*^*dsRNA*^**(H)**, *tum*^*dsRNA*^ **(I)**, or *pav*^*dsRNA*^ **(J)**. Anterior is up, Scale bar is 75μm. **(K)** Quantification of the wing area between the L3 and L4 veins as a percent of total wing area. Shown are mean and S.D., N = 4 wings). **(L)** Quantification of nuclear size and DAPI intensity of third instar larval wing disc cells from the RNAi lines in B-J. The nuclear area (blue dots) and DAPI intensity (orange dots) of single cells of the imaginal disc from the *dpp-GAL4* expressing region of the wing pouch (RFP+) were measured and divided by the mean nuclear area and DAPI intensity of cells from the wing pouch outside of the *dpp-GAL4* expressing region (RFP-) in the same wing disc (mean and S.D. for N ≥ 2 discs, and 40 cells *—p<0.05, **—p<0.01, ns—not significant relative to wild type, control cells). See S7 Fig for images.

The findings in S2 cells and tissues suggested that downregulation of an MMB transcriptome of mitotic genes promotes endoreplication. It was unclear, however, which of these downregulated genes are key for the decision to switch to endoreplication cycles. To address this question, we took an integrative genetic approach, using a collection of fly strains with GAL4-inducible UAS-dsRNAs to knock down the expression of genes that RNA-Seq had indicated were downregulated in iECs. We used an inclusive criterion and knocked down genes that were downregulated by log2 fold of at least -0.5 in both CycA and Myb dsRNA iECs, but without regard to p value (244 available strains representing 240 genes) [64] (S7 Table). We used *dpp-GAL4* to express these dsRNAs along the anterior-posterior compartment boundary of the larval wing disc, and then examined the hair pattern in the central part of adult wings between veins L3 and L4, the region that is the known fate of cells that express *dpp-GAL4* [65, 66] (Fig 5A). Each hair on the adult wing represents an actin protrusion from a single cell, and it is known that polyploidization of wing cells results in fewer and larger hairs (Fig 5B) [67–69]. As proof of principle, expression of a *UAS-CycA*^*dsRNA*^ along the A/P boundary resulted in a central stripe of longer hairs on the adult wing surface and wing margin between veins L3 and L4, with many of these cells producing clusters of multiple hairs (Fig 5C). Analysis of larval wing discs co-expressing a *UAS-mRFP* reporter showed that *dpp-GAL4*; *UAS-CycA*^*dsRNA*^ cells at the A/P compartment boundary had larger nuclei and increased DNA content compared to control cells outside of this *dpp-GAL4* stripe, confirming that the adult wing phenotype is a result of endoreplication (Fig 5C and 5L, S7B Fig). Knockdown of Myb also resulted in an increased DNA content of wing disc cells and a stripe of larger and more widely spaced adult wing hairs (Fig 5D and 5L, S7C Fig). Although both of these Myb knockdown phenotypes were less severe than that of CycA knockdown, this is likely because this Myb dsRNA is inefficient (S8 Fig). Consistent with this, raising *dpp-GAL4; UAS-Myb*^*dsRNA*^ larvae at 29 C, a temperature at which transcriptional induction by GAL4 is stronger, resulted in a more severe endoreplication phenotype that was similar to CycA (Fig 5E and 5L). Knockdown of either CycA or Myb resulted in a reduced wing surface area between wing veins L3 and L4, suggesting that growth by an increase in cell size (hypertrophy) was not able to completely recapitulate normal tissue growth by cell proliferation (Fig 5C–5E and 5K). Among the 244 strains tested, 26 resulted in lethality before adulthood, suggesting that their functions are essential (S7 Table).

Among the other 218 crosses that survived to adults, knockdown of five genes reproducibly resulted in a reduction in the area between the L3 and L4 veins and abnormal wing hairs–*aurora B* (*aurB*), *Incenp*, *Spc25*, *tumbleweed (tum)*, *and pavarroti (pav)*. Of these, three reproducibly had enlarged wing hairs and a corresponding increased DNA content of wing disc cells–*aurora B* (*aurB*), *tumbleweed (tum)*, *and pavarroti (pav)* (Fig 5F–5L, S7D–S7I Fig) [70–74]. Remarkably, all of these genes are either part of the chromosomal passenger complex (CPC) or are downstream effectors of it. The AurB kinase and INCENP are two subunits of the four-subunit CPC complex, which phosphorylates downstream targets to regulate multiple processes of mitosis and cytokinesis [75, 76]. Spc25 is a subunit of the Ndc80 outer kinetochore complex, which is phosphorylated by the CPC to regulate microtubule-kinetochore attachments [77, 78]. The Tum protein is a Rac-GAP protein that is phosphorylated by the CPC and regulates the kinesin Pav for proper cytokinesis [79]. While knockdown of any of these five genes resulted in longer hairs on the wing margin and surface, knockdown of *aurB* affected hair length primarily in the anterior half of the L3 / L4 intervein region (Fig 5F). This mild phenotype is not unexpected because the *UAS-aurB*^*dsRNA-1*^ transgene in this strain is based on a series of vectors that are not highly efficient for dsRNA expression. The stronger phenotype in the anterior could reflect the influence of patterning signals on a cells propensity to endoreplicate, although it could also be the result of different levels of *dpp-GAL4* expression and *aurB* knockdown in different cells. Expression of a more efficient *UAS-aurB*^*dsRNA-2*^ had resulted in pupal lethality before adulthood, and RT-qPCR indicated that it knocked down aurB mRNA to lower levels than *UAS-aurB*^*dsRNA-1*^ (S8 Fig). Examination of wing disc cells showed that while *UAS-aurB*^*dsRNA-1*^ induced a low level of polyploidy, *UAS-aurB*^*dsRNA-2*^ resulted in very large polyploid cells, suggesting that a strong knockdown of AurB results in high levels of endoreplication (Fig 5L, S7D and S7E Fig). All five of these MMB-regulated genes were expressed at significantly lower levels in both iECs and devECs. The combined genomic and genetic results suggest, therefore, that a dampened CycA—Myb—AurB network promotes a switch from mitotic cycles to endoreplication cycles.

### Knockdown of a CycA—Myb—AurB network induces endoreplication in ovarian follicle cells

To test whether the status of the CycA—Myb—AurB network determines the decision between mitotic and endoreplication cycles in tissues other than the wing disc, we analyzed the effects in somatic follicle cells of the ovary. These cells have specific advantages for quantifying cell cycle and cell growth. Follicle cells form a regular epithelial sheet that surrounds 15 germline nurse cells and one oocyte in each maturing egg chamber. Their regimented cell cycle programs are well characterized and coupled with stages of oogenesis, dividing mitotically during stages 1–6, undergoing three endocycles during stages 7-10A, and then selectively re-replicating genes required for eggshell synthesis during stages 10B-14 [80–82]. We induced conditional knockdown of the genes identified in the wing screen using the heat-inducible GAL4 / UAS FLP-On system, which results in clonal activation of GAL4 and induction of a *UAS-dsRNA* and a *UAS-RFP* reporter in a subset of cells [83]. This conditional knockdown also permitted an analysis of genes whose knockdown resulted in lethality in the wing screen. Three days after heat induction, we quantified the number of cells in the clone, their nuclear size, and their DNA content by measuring DAPI fluorescence. If a gene knockdown induces a switch from mitotic to endoreplication cycles, it should result in clones with fewer cells that have an increase in nuclear size and DNA content. We had shown previously shown that knockdown of *CycA* or over-expression of *Fzr* (Cdh1) induces mitotic follicle cells into precocious endocycles during early oogenesis [36, 42].

**Fig 6:**
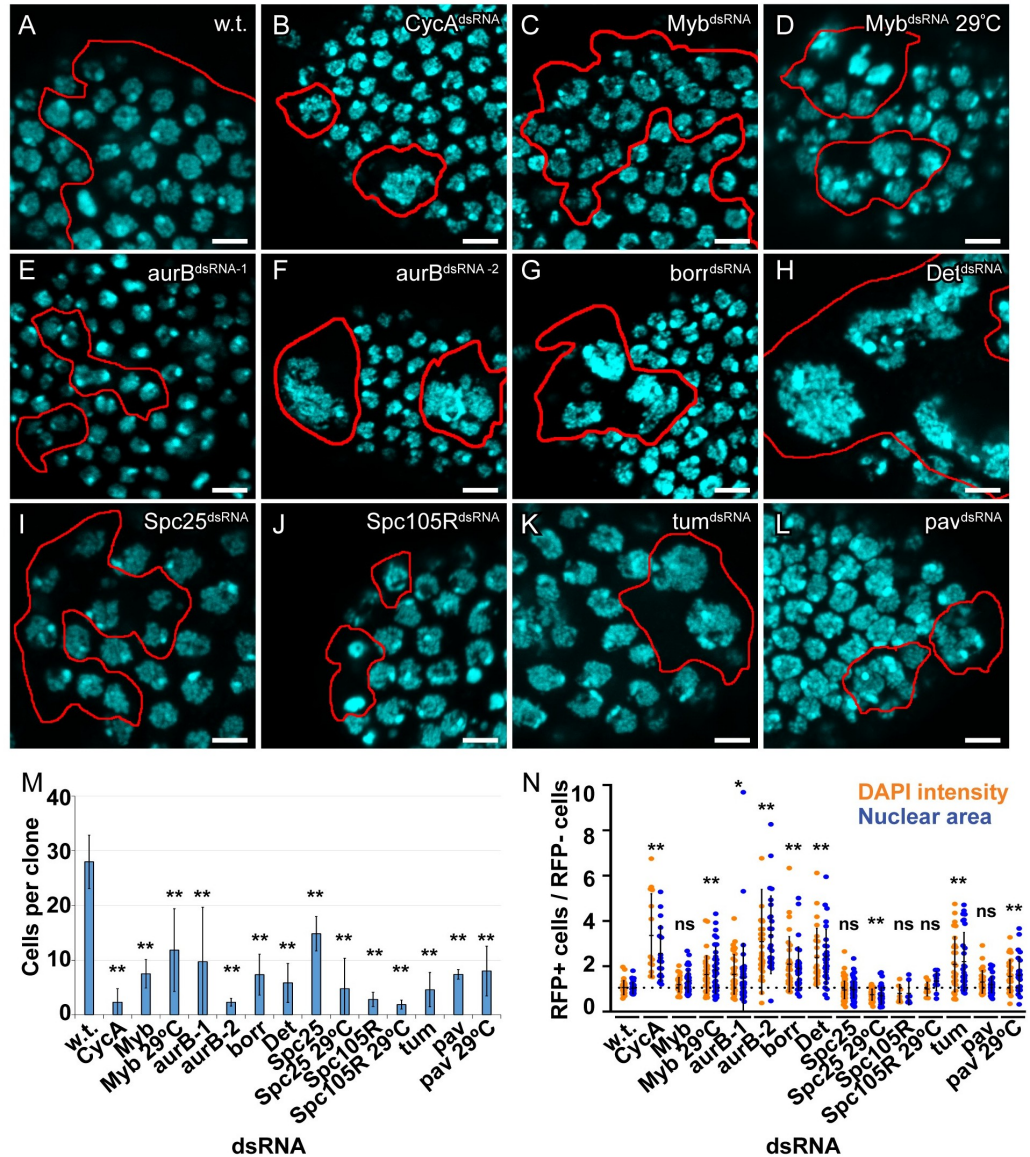
Knockdown of a CycA—Myb—AurB network induces endoreplication in ovarian follicle cells. **(A-L)** Clonal knockdown in follicle cells. FLP-On of GAL4 expression was induced by heat treatment of adult females of genotype *hsp70-FLP*, *Act>cd2>Gal4*, *UAS-mRFP* and the indicated *UAS-dsRNA*. mRFP positive clones were analyzed in stage 6 egg chambers three days later by labeling with DAPI and measuring nuclear size and total fluorescence intensity (DNA content). Red outlines in **A-L** indicate clone boundaries. **(M)** Quantification of number of cells per clone. RFP expressing cells were counted from at least four independent clones. More clones were counted in samples with fewer cells / clone (mean and S.D. for N ≥ 4 clones, **—p<0.01 relative to wild type control clones). **(N)** Quantification of nuclear area and DAPI intensity. The nuclear area (blue dots) and DAPI intensity (orange dots) of single cells in a clone (RFP+) were measured and divided by the mean nuclear area and DAPI intensity of wild type cells (RFP-) in the same egg chamber (mean and S.D. for N ≥ 3 clones, *—p<0.05, **—p<0.01, ns—not significant relative to wild type, control cells).

We analyzed clones in stage 6, the latest stage of oogenesis during which follicle cells mitotically divide. Based on the known rate of egg chamber maturation, patches of RFP+ follicle cells in these stage 6 egg chambers represent the clonal descendants of single founder follicle cells that were either transit amplifying stem cell daughters or in stage 1 egg chambers at the time of induction three days earlier. Wild type, control clones were comprised of ~28 RFP-positive cells, indicating that they had divided ~4–5 times since FLP-On in the original single founder cell (Fig 6A and 6M). FLP-On of *UAS-CycA*^*dsRNA*^ resulted in clones with only one to three cells, indicating that cell division was strongly inhibited, and that they had switched to endocycles during the first or second mitotic cycle after *CycA* knockdown (Fig 6B and 6M, Table 2). The cells in these clones had a single large nucleus with increased DNA content up to ~16C, indicating that they had endoreplicated, consistent with our previously published results (Fig 6B and 6N, Table 1) [36]. Expression of *UAS-Myb*^*dsRNA*^ resulted in some clones with reduced cell numbers and larger nuclei, suggesting that they had switched from mitotic divisions to endoreplication, but with variable expressivity among clones (Fig 6C, 6M and 6N, Table 1). A few Myb knockdown cells had two nuclei that were increased in size and DNA content, suggesting that these cells had failed cytokinesis before replicating their DNA again, a type of endomitosis. This variably expressive phenotype is likely the result of partial Myb knockdown by the inefficient *UAS-Myb*^*dsRNA*^ transgene (S8 Fig). To address this, we compared these *UAS-Myb*^*dsRNA*^ clones at 25°C to those grown at 29°C, a higher temperature that increases GAL4 activity. The clones at 29°C had a stronger phenotype, with many Myb knockdown cells having very large polyploid nuclei (Fig 6D and 6N). These results are consistent with the results in S2 cells and wing discs, and indicate that knockdown of CycA or Myb is sufficient to induce endoreplication.

**Table 2:** Summary of follicle cell clone phenotypes.

Gene dsRNA	# Cells / clone^1^	Size of nuclei	Binucleate cells / clone
CycA	↓↓↓	↑↑↑	—
Myb	↓↓	↑	↑
Myb, 29°C	↓	↑↑	↑
aurB-1	↓	↑↑	↑↑
aurB-2	↓↓↓	↑↑↑	↑
borr	↓↓	↑↑	↑
Det	↓↓	↑↑	—
Spc25	↓	↓	—
Spc25, 29°C	↓	↓	—
Spc105R	↓↓↓	↓	—
Spc105R, 29°C	↓↓↓	↓	—
pav	↓↓↓	—	↑↑↑
pav, 29°C	↓↓↓	↑↑	↑↑
tum	↓↓	↑↑	↑↑

^1^: ↓ decreased relative to control, ↑ increased relative to control, — no difference.

The combined RNA-seq and genetic screen results suggested that reduced expression of CPC subunits and other targets downstream of Myb contributes to the switch to endoreplication. Clones expressing the weaker *UAS-aurB*^*dsRNA-1*^ had only two to three cells, indicating that cell proliferation was strongly inhibited, each with variable increases in nuclear size and DNA content (Fig 6E, 6M and 6N, Table 2). A few of these cells had two nuclei of increased size and DNA content, suggesting that *UAS-aurB*^*dsRNA-1*^ impaired cytokinesis followed by endoreplication. FLP-ON expression of the stronger *UAS-aurB*^*dsRNA-2*^ in follicle cells resulted in clones composed of only one to two cells, each with a single, large, polyploid nucleus (Fig 6F, 6M and 6N, Table 2). Many of these nuclei were multi-lobed, with connected chromatin masses composed of large chromosomes that appeared polytene (Fig 6F). These results suggest that mild knockdown of AurB results in cytokinesis failure, whereas a stronger knockdown results in a failure to segregate chromosomes and cytokinesis, followed by endoreplication.

To further test whether reduced CPC activity induces endoreplication, we knocked down expression of its other three subunits: Incenp, Borealin-related (Borr), and Deterin (Det) (fly Survivin ortholog), all of which were expressed at lower levels in iECs and salivary gland devECs (S3 and S5 Tables) [72, 84, 85]. Expression of *UAS-Incenp*^*dsRNA*^ did not reduce the number of cells per clone nor increase DNA content, consistent with its lack of effect in the wing discs, an uninformative negative result because this UAS transgene is optimized for expression in the germline but expressed poorly in the soma (Table 2). Knockdown of the other CPC subunits, Borr and Det, had resulted in lethality in the wing screen, whereas their conditional knockdown in follicle cells resulted in clones with very few cells, each with large, polyploid nuclei (Fig 6G, 6H, 6M and 6N, Table 2). As a further test of the importance of the CPC, we knocked down *aurB* in S2 cells, and compared the effect of its knockdown to another mitotic kinase gene, *polo*. Similar to wing and ovarian follicle cells, knockdown of *aurB* in S2 cells resulted in endoreplication, whereas knockdown of *polo* resulted in a mitotic arrest (S9A and S9B Fig). These results indicate that reduction of CPC activity is sufficient to switch cells from mitotic cycles to endoreplication cycles.

### Knockdown of cytokinetic, but not kinetochore, proteins induces endoreplication in follicle cells

We then addressed which processes downstream of the CPC are crucial for the mitotic cycle versus endoreplication cycle decision in follicle cells [76]. The genes *Spc25*, *tum*, *and pav* encode proteins that function downstream of the CPC [70, 79, 86, 87]. All three of these genes are regulated by the MMB, were expressed at lower levels in iECs and devECs, and were recovered in the wing screen (Figs 4C and 5H–5J). Knockdown of the kinetochore protein Spc25 in follicle cells strongly inhibited cell division and resulted in fewer cells per clone (Fig 6I, 6M and 6N). However, the nuclear size and DNA content of these cells were not increased, indicating that endoreplication was not induced, consistent with results from the wing (Figs 5L and 6I and 6N, Table 2). To further evaluate kinetochore proteins downstream of the CPC, we knocked down Spc105R, a kinetochore protein important for microtubule attachment and the spindle assembly checkpoint (SAC) [88]. Clonal knockdown of Spc105R strongly inhibited cell proliferation, and induced pyknotic nuclei indicative of programmed cell death, but did not result in an increase in DNA content (Fig 6J, 6M and 6N, Table 2). Growing the Spc25 and Spc105R clones at 29°C to enhance knockdown resulted in fewer cells per clone, and more nuclei that appeared pyknotic, but again did not result in enlarged nuclei (Fig 6M and 6N). Thus, despite a strong mitotic arrest phenotype, knockdown of these two kinetochore proteins downstream of the CPC did not result in endoreplication. Knockdown of the cytokinesis proteins Tum or Pav resulted in many fewer cells per clone, with many binucleate, indicating that cytokinesis was inhibited (Fig 6K–6M, Table 2). Unlike kinetochore protein knockdown, however, the binucleate Tum knockdown cells had a significant increase in both nuclear size and DNA content per nucleus (mean ~2 fold, max ~4 fold increase), indicating that they had endoreplicated after a failure of cytokinesis, consistent with the results from the wing disc (Figs 5K, 6K and 6N, Table 2). While some Pav knockdown cells clearly had larger polyploid nuclei (~2 fold), the average was not significantly different from control cell populations, unlike the results from wings where Pav knockdown induced significant polyploidy (Figs 5L, 6L, 6M and 6N, Table 2). Stronger knockdown of *pav* at 29°C, however, did result in a significant increase in nuclear area and DAPI intensity, consistent with the interpretation that these cells have undergone endoreplication (Fig 6N). Thus, inhibition of cytokinetic, but not kinetochore, proteins downstream of the CPC induces an endoreplication cycle. All together, these results suggest that the status of a CycA—MMB—AurB network determines the choice between mitotic and endoreplication cycles.

## Discussion

We have investigated how the cell cycle is remodeled when mitotic cycling cells switch into endoreplication cycles, and how similar this remodeling is between devECs and experimental iECs. We have found that repression of a CycA—Myb—AurB mitotic network promotes a switch to endoreplication in both devECs and iECs. Although a dampened E2F1 transcriptome of S phase genes is a common property of devECs in flies and mice, we found that repression of the Myb transcriptome is sufficient to induce endoreplication in the absence of reduced expression of the E2F1 transcriptome. Knockdown of different components of the CycA-Myb-AurB network resulted in endoreplication cycles that repressed mitosis to different extents, which suggests that regulation of different steps of this pathway may explain the known diversity of endoreplication cycles *in vivo*. Overall, these findings define how cells either commit to mitosis or switch to different types of endoreplication cycles, with broader relevance to understanding the regulation of these variant cell cycles and their contribution to development, tissue regeneration, and cancer.

**Fig 7:**
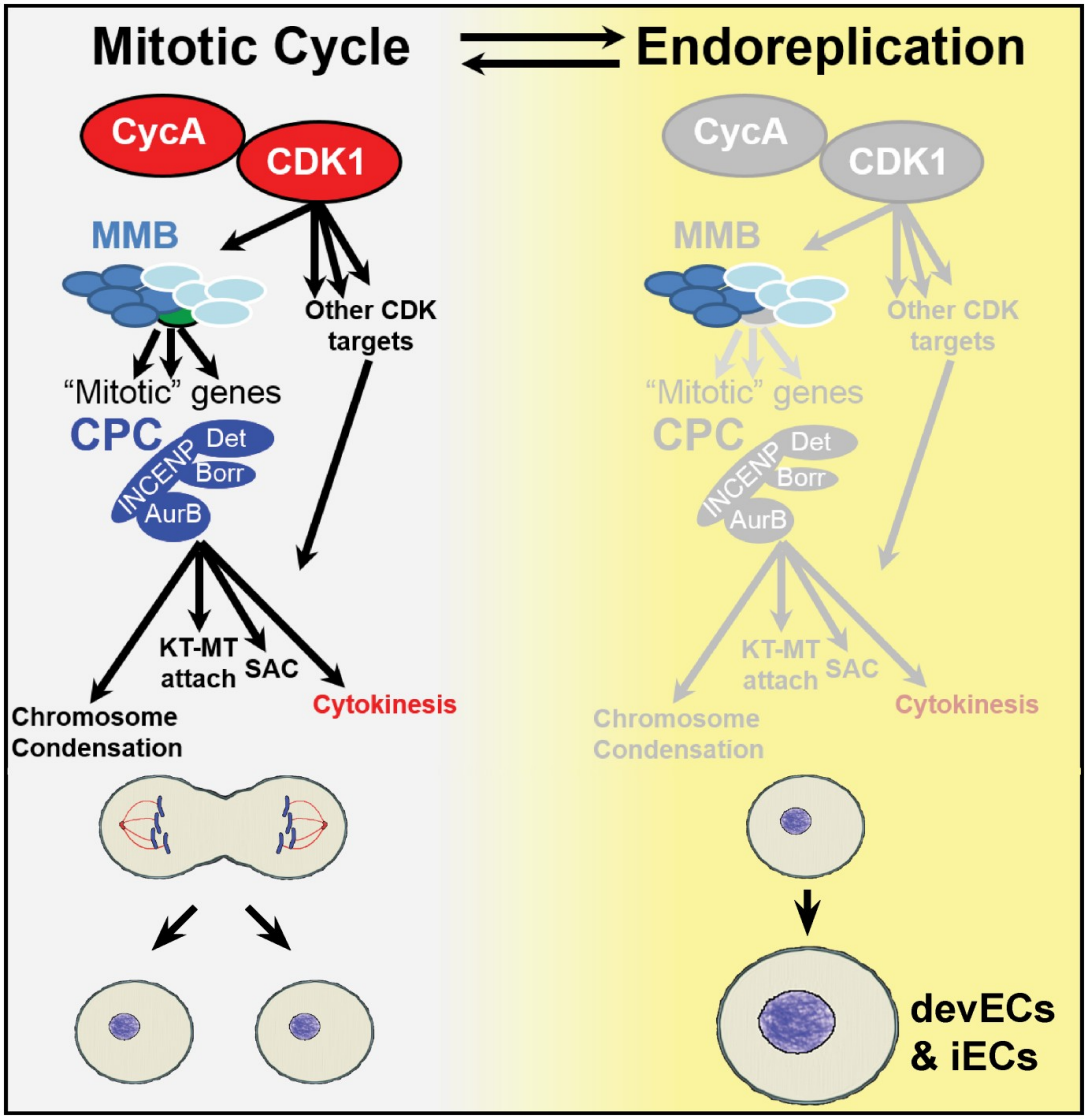
Model: The CycA—Myb—AurB network regulates the choice between cell cycle programs. Depicted are two alternative cell cycle programs, the mitotic cycle (left), and endoreplication cycle (right, yellow). During mitotic cycles, CycA / CDK1 activates the Myb-MuvB (MMB) to induce transcription of multiple genes with mitotic functions (“mitotic” genes). Among these are the subunits of the chromosome passenger complex (CPC), which phosphorylates multiple targets to regulate chromosome condensation, kinetochore-microtubule (KT-MT) attachment, the spindle assembly checkpoint (SAC), and cytokinesis. Our findings suggest that CycA / CDK1, MMB, and the CPC are key nodes of this mitotic network whose repression promotes a transition to endoreplication in both iECs and devECs. See text for further details.

Our findings indicate that the status of the CycA—Myb—AurB network determines the choice between mitotic or endoreplication cycles (Fig 7). These proteins are essential for the function of their respective protein complexes: CycA activates CDK1 to regulate mitotic entry, Myb is required for transcriptional activation of mitotic genes by the MMB transcription factor complex, and AurB is the kinase subunit of the four-subunit CPC. While each of these complexes were previously known to have important mitotic functions, our data indicate that they are key nodes of a network whose activity level determines whether cells switch to the alternative growth program of endoreplication (Fig 7). Our results are consistent with previous evidence in several organisms that lower activity of the Myb transcription factor results in polyploidization, and further shows that repressing the function of the CPC and cytokinetic proteins downstream of Myb also promotes endoreplication [13, 16, 23, 89]. Importantly, our genetic evidence indicates that not all types of mitotic inhibition result in a switch to endoreplication. For example, knockdown of the Spc25 and Spc105R kinetochore proteins or the Polo kinase resulted in a mitotic arrest, not a switch to repeated endoreplication cycles. These observations are consistent with CycA / CDK, MMB, and the CPC playing principal roles in the mitotic network hierarchy and the decision to either commit to mitosis or switch to endoreplication cycles.

While knockdown of different proteins in the CycA-Myb-AurB network were each sufficient to induce endoreplication cycles, these iEC populations had different fractions of cells with multiple nuclei diagnostic of an endomitotic cycle. Knockdown of cytokinetic genes *pav and tum* resulted in the highest fraction of endomitotic cells, followed by the CPC subunits, then *Myb*, and finally *CycA*, with knockdown of this cyclin resulting in the fewest endomitotic cells. These results suggest that knocking down genes higher in this branching mitotic network (e.g. CycA) inhibits more mitotic functions and preferentially promotes G / S endocycles that skip mitosis, whereas inhibition of functions further downstream in the network promote endomitosis (Fig 7). Moreover, we found that different levels of CPC function also resulted in different subtypes of endoreplication. Strong knockdown of AurB inhibited chromosome segregation and cytokinesis resulting in cells with a single polyploid nucleus, whereas a mild knockdown resulted in successful chromosome segregation but failed cytokinesis, suggesting that cytokinesis requires more CPC function than chromosome segregation. It thus appears that different thresholds of mitotic function result in different types of endoreplication cycles. This idea that endomitosis and endocycles are points on an endoreplication continuum is consistent with our evidence that treatment of human cells with low concentrations of CDK1 or AurB inhibitors induces endomitosis, whereas higher concentrations induce endocycles [28]. Our results raise the possibility that in tissues of flies and mammals both conditional and developmental inputs may repress different steps of the CycA—Myb—AurB network to induce slightly different types of endoreplication cycles that partially or completely skip mitosis [5, 90]. Together, our findings show that there are different paths to polyploidy depending on both the types and degree to which different mitotic functions are repressed.

Our findings are relevant to the regulation of periodic MMB transcription factor activity during the canonical mitotic cycle. Knockdown of CycA compromised MMB transcriptional activation of mitotic gene expression, and their physical association suggests that the activation of the MMB by CycA may be direct. The MMB-regulated mitotic genes were expressed at lower levels in CycA iECs, even though Myb protein levels were not reduced. This result is consistent with the hypothesis that CycA / CDK phosphorylation of the MMB is required for its induction of mitotic gene expression. Moreover, misexpression of Myb in CycA knockdown follicle cells did not prevent the switch to endoreplication, further evidence that CycA / CDK is required for MMB activity and mitotic cycles (S10 Fig). While the dependency of the MMB on CycA was not previously known in *Drosophila*, it was previously reported that in human cells CycA / CDK2 phosphorylates and activates human B-Myb in late S phase, and also triggers its degradation [53, 91]. While further experiments are needed to prove that CycA / CDK regulation of the MMB is direct, interrogation of the results of multiple phosphoproteome studies using iProteinDB indicated that *Drosophila* Myb protein is phosphorylated at three CDK consensus sites including one, S381 that is of a similar sequence and position to a CDK phosphorylated site on human B-Myb (T447) [92, 93]. We favor the hypothesis that it is CycA complexed to CDK1 that regulates the MMB because, unlike human cells, in *Drosophila* CycA / CDK2 is not required for S phase, and Myb is degraded later in the cell cycle during mitosis [45, 94]. Moreover, it is known that mutations in CDK1, but not CDK2, induce endocycles in *Drosophila*, mouse, and other organisms [37, 95]. A cogent hypothesis is that CycA / CDK1 phosphorylates Myb, and perhaps other MMB subunits, to stimulate MMB activity as a transcriptional activator of mitotic genes, explaining how pulses of mitotic gene expression are integrated with the master cell cycle control machinery (Fig 7). It remains formally possible, however, that both CycA / CDK2 and CycA / CDK1 activate the MMB in *Drosophila*. The early reports that CycA / CDK2 activates B-Myb in human cells were before the discovery that it functions as part of the MMB and the identification of many MMB target genes, and further experiments are needed to fully define how MMB activity is coordinated with the central cell cycle oscillator in fly and human cells [17, 19, 24, 26].

We experimentally induced endocycles by knockdown of CycA to mimic the repression of CDK1 that occurs in devECs. Our data revealed both similarities and differences between these experimental iECs and devECs. Both iECs and SG devECs had a repressed CycA—Myb—AurB network of mitotic genes. In contrast, only devECs had reduced expression of large numbers of E2F1-dependent S phase genes, a conserved property of devECs in fly and mouse [10, 13–15]. In CycA iECs, many of these key S phase genes were not downregulated, including *Cyclin E*, *PCNA*, and subunits of the pre-Replicative complex, among others. This difference between CycA dsRNA iECs and SG devECs indicates that repression of these S phase genes is not essential for endoreplication. In fact, none of the E2F1 -dependent S phase genes were downregulated in Myb dsRNA iEC. Instead, the 12 E2F1-dependent genes that were commonly downregulated in Myb dsRNA iEC, CycA dsRNA iEC, and SG devEC all have functions in mitosis (Table 1). These 12 mitotic genes are, therefore, dependent on both Myb and E2F1 for their expression, including the cytokinetic gene *tum* whose knockdown induced endomitotic cycles. This observation leads to the hypothesis that downregulation of the E2F transcriptome in fly and mouse devECs may serve to repress the expression of these mitotic genes, and that the repression of S phase genes is a secondary consequence of this regulation. These genomic data, together with our genetic evidence in S2 cells and tissues, indicates that in *Drosophila* the repression of the Myb transcriptome is sufficient to induce endoreplication without repression of the E2F1 transcriptome. The observation that both CycA^dsRNA^ iECs and devECs both have lower CycA / CDK activity, but differ in expression of E2F1 regulated S phase genes, also implies that there are CDK-independent mechanisms by which developmental signals repress the E2F1 transcriptome in devECs.

Our results have broader relevance to the growing number of biological contexts that induce endoreplication. Endoreplicating cells are induced and contribute to wound healing and regeneration in a number of tissues in fly and mouse, and, depending on cell type, can either inhibit or promote regeneration of the zebrafish heart [27, 30–32]. An important remaining question is whether these iECs, like experimental iECs and devECs, have a repressed CycA—Myb—AurB network. If so, manipulation of this network may improve regenerative therapies. In the cancer cell, evidence suggests that DNA damage and mitotic stress, including that induced by cancer therapies, can switch cells into an endoreplication cycle [5, 41, 96, 97]. These therapies include CDK and AurB inhibitors, which induce human cells to polyploidize, consistent with our fly data that CycA / CDK and the CPC are key network nodes whose repression promotes the switch to endoreplication [75, 98]. Upon withdrawal of these inhibitors, transient cancer iECs return to an error-prone mitosis that generates aneuploid cells, which have the potential to contribute to therapy resistance and more aggressive cancer progression [28, 99–101]. Our finding that the Myb transcriptome is repressed in iECs opens the possibility that these mitotic errors may be due in part to a failure to properly orchestrate a return of mitotic gene expression. Understanding how this and other networks are remodeled in polyploid cancer cells will empower development of new approaches to prevent cancer progression.

## Materials and methods

### *Drosophila* genetics

*Drosophila* strains were obtained from the Bloomington Stock Center (BDSC, Bloomington, IN), or the Vienna Drosophila Resource Center (VDRC, Vienna Austria). The *UAS-mRFP-Myb* strain was kindly provided by Dr. Joe Lipsick. *Drosophila* were raised on BDSC standard cornmeal medium at 25°C unless otherwise indicated. For the genetic screen of Fig 4, fly strains with *UAS-dsRNA* transgenes were made by the Drosophila RNAi Screening Center (DRSC) and provided by the BDSC. These strains were crossed to *dpp-Gal4*, *UAS-mRFP* and multiple progeny of each cross were scored for their adult wing phenotype. Specific details about genotypes and strain numbers can be found in S7 and S8 Tables.

### Cell culture and dsRNA treatment

S2 cells were grown at 25°C in M3 + BPYE medium supplemented with 10% Fetal Bovine Serum as described [102]. iECs were supplemented with an additional 2% Fetal Bovine Serum (12% final). Cell proliferation in S2 Fig was quantified by counting cells using a hemocytometer. For RNAi, S2 cells were treated with the indicated dsRNA for 1 hour in serum free medium, followed by culturing for 96 hours at 25°C, as indicated above, and then analyzed as indicated below.

### Flow cytometry

After dsRNA treatment, S2 cells were harvested in PBS and fixed in ethanol. After fixation, cells were incubated in propidium iodide (20 μg/ml) supplemented with RNaseA (250 μg/ml) at 37°C for 30 minutes. Flow cytometry was performed using an LSRII (BD Biosciences) and data were analyzed with Flowjo v7.6.5 software.

### SDS-PAGE and western blotting

Protein extracts were made from S2 cells using a non-denaturing lysis buffer (25mM Tris, pH 7.5, 150mM NaCl, 5mM EDTA, 1% IGEPAL (Sigma-Aldrich), 5% glycerol, complete protease inhibitor cocktail (Sigma-Aldrich), PhosSTOP (Sigma-Aldrich)) and homogenizing the cells on ice. Absolute protein levels were determined by Bradford assays. At least 20 μg protein was separated by SDS-PAGE, electrophoretically transferred to PVDF membranes, and blotted using the following antibodies: anti-Cyclin A (A12, DSHB, concentrate) at 1:1000, anti-Cyclin B (F2F4, DSHB supernatant) at 1:100, anti-HA (Y11, Santa Cruz) at 1:1000, anti-Myb (D3R, provided by J. Lipsick) at 1:1000, anti-Tubulin (E7, DSHB, concentrate) at 1:1000. Blots were labeled with HRP conjugated secondary antibodies and developed using Super Signal West Pico substrate (Thermo Scientific).

### Immunoprecipitation

*Hsp70-Gal4*, *UAS-mRFP or Hsp70-Gal4*, *UAS-mRFP-Myb* flies were crossed to *UAS-CycA* (Fig 3C) or *UAS-CycA-HA* (Fig 3C’) flies. Larvae were heat treated three times at 37°C for 30 minutes over 1.5 days beginning in 2^nd^ instar, and protein extracts made from early 3^rd^ instar larvae by homogenizing in non-denaturing lysis buffer (indicated above) for 1 hour after the final heat treatment. Lysate was quantified using Bradford assays to normalize total protein content among samples. In Fig 3C, extracts were immunoprecipitated using highly-efficient RFP-Trap (Chromotek) single-chain nanobodies made in camelids and conjugated to agarose beads. In Fig 3C’, extracts were immunoprecipitated with anti-HA (F7, Santa Cruz) or normal mouse serum on Protein G Agarose (Invitrogen). Western blots of input and IP were incubated with antibodies against *Drosophila* Myb (gift of J. Lipsick), Cyclin A (DHSB), DsRed (Takara), and HA (Santa Cruz).

### Labeling and immunofluorescent microscopy of S2 cells

In Fig 2, S2 cells were treated with dsRNA for 96 hours at 25°C, replated on poly-D-lysine coated chamber slide, and allowed to settle for 16–18 hours. Cells were then incubated in EdU (20μM) for 2 hours at 25°C followed by click-it fluorescent labeling according to the manufacturer’s (Invitrogen) protocol. These cells were then labeled with antibodies against (pH3) (Millipore, 06–570) and appropriate fluorescent secondary antibodies. Cells were stained with DAPI (0.5μg/ml) and imaged on a Leica SP5 confocal or Leica DMRA2 fluorescent microscope. The fraction of EdU and pH3 labeled cells and nuclear area were quantified using ImageJ v1.50b software (https://imagej.nih.gov/ij/).

### Labeling and immunofluorescent microscopy of wing imaginal discs

For S7 Fig, wing imaginal discs were dissected from 3^rd^ instar larvae and labeled with antibodies against DsRed (Takara) followed by labelling with anti-rabbit Alexa Fluor 568 (Thermo Fisher). Cells were stained with DAPI (0.5μg/ml) and imaged on a Leica SP5 confocal or Leica DMRA2 fluorescent microscope. Nuclear area and DAPI fluorescence was measured with ImageJ. Nuclear area and DAPI fluorescence of GAL4-expressing, DsRed-positive cells within the wing pouch was normalized to that of DsRed-negative cells in the wing pouch of the same discs.

### Generation of iEC ovary clones, labeling, and immunofluorescent microscopy

*Hsp70-FLP;Act>cd2>Gal4*, *UAS-mRFP* was crossed to different *UAS-dsRNA* fly strains. Well-fed 3–5 day old adult G1 females were heat induced at 37°C for 30 minutes and allowed to recover for three days before ovaries were dissected, and labeled with anti-dsRed (Takara) and counterstained with DAPI as previously described [36]. Cell clones in stage 6 egg chambers were imaged on a Leica SP5 confocal and Leica DMRA widefield epifluorescent microscope. Cell number was quantified by counting RFP+ cells. The area and total DAPI fluorescence of nuclei within individual cells of a clone (RFP+) were measured using ImageJ and normalized to the average of wild type cells outside of the clone (RFP-) in the same egg chamber.

### RT-qPCR

mRNA for RT-qPCR was isolated by TRIzol (Invitrogen) according to the manufacturer’s instructions. cDNA was generated using the Superscript III kit (Invitrogen). qPCR was performed using Brilliant III Ultra-Fast SYBR Green qPCR Master Mix (Agilent Technologies) and the primers indicated in S8 Table. Act5C was amplified as an internal reference control. Data were analyzed using LinRegPCR software (ver. 2016.2) the Pfaffl method to determine relative transcript levels [103, 104].

For S2 cell RT-qPCR, RNA was isolated 96 hours after dsRNA knockdown or control GFP dsRNA. Each assay was performed with technical duplicates and biological triplicates.

For quantification of knockdown in discs in S8 Fig, *hsp70-GAL4; UAS-dsRNA* and control *hsp70-GAL4* only larvae were heat treated twice at 37 C for ½ hour over one day, and mRNA was isolated from 3^rd^ instar discs ½ hour after the second heat shock and RT-qPCR performed as described above. Reactions were done in technical and biological duplicates. mRNA levels in the knockdown strains were normalized to levels in the hsp70-GAL4 control strain.

### Statistical analysis

Statistical analysis of Figs 1B, 1C, 2B, 2G, 2H, 3B and 6M, S9A and S9B Fig were performed using two-tailed Student’s *t* tests using Microsoft Excel (version 15.0.4753.1000). For Fig 2F and S10 Fig a two-tailed Welch’s *t* test was performed using GraphPad Prism (version 7.04), For Figs 5L, and 6N GraphPad Prism (version 7.04) was used to perform a one-way ANOVA with a two-stage linear step-up procedure of Benjamini, Krieger and Yekutieli post-hoc test [105] to assess statistical difference between control clones and the indicated dsRNA clones.

### RNA-Seq analysis

For RNA-Seq of S2 cells, RNA was prepared from three biological replicates of CycA dsRNA, Myb dsRNA, and GFP dsRNA treated cells. For tissues, RNA was prepared from salivary glands (SG) or brains plus imaginal discs (B-D) from the same feeding early third instar larvae in three biological replicates, as previously described [13]. TruSeq Stranded mRNA Libraries (Illumina) were prepared by the Center for Genomics and Bioinformatics (CGB) of Indiana University according to manufacturer’s protocol. Multiplex sequencing barcodes from TruSeq RNA Single Indexes set A or B (Illumina) were added to the libraries during construction. The barcoded libraries were cleaned by double side beadcut with AMPure XP beads (Beckman Coulter), verified using Qubit3 fluorometer (ThermoFisher Scientific) and 2200 TapeStation bioanalyzer (Agilent Technologies), and then pooled. The pool was sequenced on NextSeq 500 (Illumina) with NextSeq75 High Output v2 kit (Illumina). Single-end 75 bp read sequences were generated. The read sequences were de-multiplexed using bcl2fastq (software versions 1.4.1.2, 1.4.1.2, and 2.1.0.31 for GSF1389, GSF1471, GSF1611).

### Bioinformatics

Read quality was checked with FastQC v0.11.5 [106], and reads were then mapped against the Dmel R6.23 genome assembly and annotation using STAR v2.6.1a [107]. Mapped fragments were assigned to exons via the featureCounts function of the Rsubread v1.24.2 bioconductor package [108], and various pseudogenes and ncRNAs were excluded. Differential gene expression between samples was calculated using DESeq2 v1.14.1 [109]. Gene lists derived from RNA-Seq data sets were categorized as upregulated (Log2 fold-change ≥ 0.5 with an FDR adjusted p ≤ 0.05) or downregulated (Log2 fold-change ≤ -0.5 with an FDR adjusted p ≤ 0.05) [59]. Human ortholog information and DIOPT scores were downloaded from FlyBase on 09-11-2018 [110] and GO terms were retrieved using the Bioconductor package AnnotationHub v2.12.0 with a snapshot date of 04-30-2018 [111]. GO enrichment analysis was performed and plots were generated using clusterProfiler v3.8.1 [112]. The comparisons between the differentially expressed genes in the RNA-seq and the accompanying Venn diagrams were created using custom scripts and the R library VennDiagram [113].

Permutation testing was used to calculate *p*-values and fold enrichment of the DE gene double overlap between CycA iEC and Myb iEC or triple overlap among CycA iEC, Myb iEC and Sg devEC relative to chance (S3 Fig) [114]. Briefly, either two or three random gene sets were sampled (for the double and triple overlap sets respectively), with total genes sampled equal to the number of DE genes observed for those samples, and the number of overlapping genes between the sampled sets was recorded. This randomization sampling process was repeated 100,000 times. The *p*-values were calculated by finding the number of permutation samples that resulted in an overlapping number of genes greater than or equal to the observed number of overlapping genes plus one, over the number of permutation samples plus one. For the enrichment plot of S3 Fig, each observed overlap value was converted to fold difference relative to the sampled overlap values, and the median, 5% and 95% quantiles are shown.

## Supporting information

S1 Data **Legend for S1–S5 Tables: FBgn**—Flybase IDs. **FDR adjusted p value (B-H)**—p value adjusted for **F**alse **D**iscovery **R**ate using the **B**enjamini-**H**ochberg method (1). Light green highlight—Gene is upregulated for the indicated comparison. Light red highlight—Gene is downregulated for the indicated comparison. Yellow highlight—Not differentially expressed for the indicated comparison. **“Human Ortholog?”**–Proposed human ortholog based on a DIOPT score of at least 3. Blank columns do not necessarily indicate that there is no ortholog, rather a blank column indicates that there aren’t sufficient data to make a high-confidence determination. **DIOPT score**—The number indicates the agreement in the ortholog call between multiple databases when the indicated gene was queried. Higher number indicate higher confidence (2).
(DOCX)

S1 Table Differentially expressed genes in CycA dsRNA iECs. (XLSX)

S2 Table Differentially expressed genes in Myb dsRNA iECs. (XLSX)

S3 Table Differentially expressed (DE) genes shared by CycA dsRNA iECs and Myb dsRNA iECs. (XLSX)

S4 Table Mitotic genes from enriched GO categories that are downregulated in iECs and devECs. (XLSX)

S5 Table Differentially expressed (DE) genes shared by CycA dsRNA iECs, Myb dsRNA iECs and salivary gland devECs. (XLSX)

S6 Table Meta-analysis of RNA-Seq data for E2F1 regulated genes (from Dimova et al. 2003)(3). (XLSX)

S7 Table Results of RNAi wing screen. Stock#—Bloomington Drosophila Stock Center (BDSC) Stock number. Light blue—positive hit in the wing screen. Reduced size of L3-L4 region, increased bristle size. Orange—Lethal. No adult flies after expression of the indicated dsRNA.
(XLSX)

S8 Table Full list of fly strains and primers used. Stock#–Bloomington Drosophila Stock Center (BDSC) Stock number.
(XLSX)

S1 Fig Knockdown of CycB does not induce endoreplication. S2 cells were treated with CycB dsRNA. **(A)** qRT-PCR quantification of CycB transcript in CycB dsRNA versus GFP dsRNA control cells. **(B)** Quantification of flow cytometry data for ploidy classes in GFP dsRNA and CycB dsRNA cells (mean and S.D. for N = 2).
(TIF)

S2 Fig Knockdown of CycA or Myb inhibits cell proliferation. 500,000 cells were plated and treated with the indicated dsRNAs. The cells were counted once every 24h for 7 days (mean and S.D. for N = 3).
(TIF)

S3 Fig Statistical analysis of DE gene overlap between populations of endoreplicating cells. Permutation testing was used to calculate *p*-values and fold enrichment of the pairwise overlap between CycA dsRNA iECs and Myb dsRNA iECs (A), and three-way overlap among CycA dsRNA iECs, Myb dsRNA iECs, and Salivary Gland devECs (B), relative to chance (4). The graph shows the fold difference between the observed overlaps and those predicted by 100,000 iterations of random sampling values based on DE gene numbers. The vertical bar represents the median, and the extent of the boxes are the 5% and 95% quantiles (p< 1 x 10^−5^ for all comparisons).
(TIF)

S4 Fig CycA dsRNA iECs and Myb dsRNA iECs have increased expression of genes involved in development. Biological Process (BP) Gene Ontology (GO) category analysis was performed on genes that were upregulated at least Log2FC 0.5, with an FDR corrected q <0.05 in both the CycA dsRNA, and Myb dsRNA iECs relative to GFP dsRNA treated cells. The graph shows number of genes in the top 20 GO categories that were significantly enriched in both iEC types with color coding indicating FDR corrected q value for that class.
(TIF)

S5 Fig CycA dsRNA iECs and Myb dsRNA iECs have decreased expression of genes required for mitosis. BP GO category analysis was performed on genes that were downregulated at least Log2FC -0.5, with an FDR corrected q <0.05 in both the CycA dsRNA, and Myb dsRNA iECs relative to GFP dsRNA treated cells. The graph shows number of genes in the top 20 GO categories that were significantly enriched in both iEC types with color coding indicating FDR corrected q value for that class.
(TIF)

S6 Fig iECs and devECs have decreased expression of Myb-induced genes that are required for mitosis. Comparison of RNA-Seq results for iEC in culture and devEC in salivary glands. BP GO category analysis was performed on genes that were downregulated at least Log2FC -0.5, with an FDR of <0.05 in the CycA dsRNA, and Myb dsRNA iECs relative to GFP dsRNA treated cells and the salivary gland endocycling vs Brain-disc tissues. The graph shows the top 20 BP GO categories that were significantly enriched in the overlap of CycA dsRNA iECs, Myb dsRNA iECs, and salivary gland devECs.
(TIF)

S7 Fig Knockdown of individual members of the CycA-Myb-aurB network is sufficient to induce endoreplication in wing imaginal discs. Wing imaginal discs corresponding to dpp-GAL4 / UAS-dsRNA wing screen genotypes indicated in Fig 5. Red outlines indicate the border of the mRFP expression that corresponds to dpp-GAL4 expression. (A) A control wild type (w.t.) wing disc from a *dpp-GAL4*, *UAS-mRFP*; *UAS-GFP* animal. (B) A wing disc from a *dpp-GAL4*, *UAS-mRFP; UAS-CycA*^*dsRNA*^ animal. Note the larger nuclei within the red border compared to cells outside. (C-I) Wing discs after expression of *UAS-Myb*^*dsRNA*^ (C), *AurB*^*dsRNA-1*^ (D), *AurB*^*dsRNA-2*^ (E), *Incenp*^*dsRNA*^ (F), *Spc25*^*dsRNA*^(G), *tum*^*dsRNA*^ (H), or *pav*^*dsRNA*^ (I). Scale bars are 20μM.
(TIF)

S8 Fig RT-qPCR quantification of RNAi knockdown in larval discs. RT-qPCR quantification of the indicated transcripts in imaginal discs from different UAS-dsRNA strains normalized to that in wild type control discs. Each value on the X axis indicates both the dsRNA strain and the transcript measured after induction with a heat inducible GAL (N = 2).
(TIF)

S9 Fig Knockdown of aurB induces endoreplication whereas knockdown of polo induces a mitotic arrest in S2 cells. (A) Flow cytometry of DNA content in propidium iodide labeled S2 cells 96 hours after treatment with either GFP dsRNA (control), aurB dsRNA or polo dsRNA. (B) Quantification of EdU and pH3 labeling in cells after treatment with the indicated dsRNAs (mean and S.E.M. for N = 3, *—p < 0.05, ** p < 0.01, ns—not significant).
(TIF)

S10 Fig Myb over-expression does not inhibit endoreplication after CycA knockdown. Induction of endoreplication by knockdown of CycA is not suppressed by overexpressing Myb. Quantification of nuclear area of ovary follicle cells in stage 6 egg chambers after heat inducing the following genotypes: 1) *UAS-GFP/+; Hsp70-GAL4*, *UAS-mRFP/+*, 2) *UAS-GFP/+; Hsp70-GAL4*, *UAS-mRFP-Myb /+*, 3) *(UAS-CycA dsRNA/+; Hsp70-GAL4*, *UAS-mRFP / +*, and 4) *UAS-CycA dsRNA/+; Hsp70-GAL4*, *UAS-mRFP-Myb / +*. Each dot represents the nuclear area of a single cell divided by the mean area of controls (machine units). Mean and S.D. N≥5 egg chambers, n≥100 cells, ns—not significant).
(TIF)
