# Supplementary material for: A Cyclin A—Myb-MuvB—Aurora B network regulates the choice between mitotic cycles and polyploid endoreplication cycles
Source: PLoS Genet. 2019 Jul 10;15(7):e1008253. doi: 10.1371/journal.pgen.1008253 (PMC6645565; doi:10.1371/journal.pgen.1008253)
Supplement: S1 Data — Legend for S1–S5 Tables: FBgn—Flybase IDs. FDR adjusted p value (B-H)—p value adjusted for False Discovery Rate using the Benjamini-Hochberg method (1). Light green highlight—Gene is upregulated for the indicated comparison. Light red highlight—Gene is downregulated for the indicated comparison. Yellow highlight—Not differentially expressed for the indicated comparison. “Human Ortholog?”–Proposed human ortholog based on a DIOPT score of at least 3. Blank columns do not necessarily indicate that there is no ortholog, rather a blank column indicates that there aren’t sufficient data to make a high-confidence determination. DIOPT score—The number indicates the agreement in the ortholog call between multiple databases when the indicated gene was queried. Higher number indicate higher confidence (2). (DOCX) [file pgen.1008253.s001.docx]

**Supplemental Data**

**Legend for Tables S1-S5:**

**FBgn** – Flybase IDs

**FDR adjusted p value (B-H)** – p value adjusted for **F**alse **D**iscovery **R**ate using the **B**enjamini-**H**ochberg method (1)

Light green highlight – Gene is upregulated for the indicated comparison

Light red highlight – Gene is downregulated for the indicated comparison

Yellow highlight – Not differentially expressed for the indicated comparison
**“Human Ortholog?”** – Proposed human ortholog based on a DIOPT score of at least 3. Blank columns do not necessarily indicate that there is no ortholog, rather a blank column indicates that there aren’t sufficient data to make a high-confidence determination.
**DIOPT score –** The number indicates the agreement in the ortholog call between multiple databases when the indicated gene was queried. Higher number indicate higher confidence (2).

**Table S1 – Differentially expressed genes in CycA dsRNA iECs**

**Table S2 – Differentially expressed genes in Myb dsRNA iECs**

**Table S3 –Differentially Expressed (DE) genes shared by CycA dsRNA iECs and Myb dsRNA iECs**

**Table S4 – Mitotic genes from enriched GO categories that are downregulated in iECs and devECs**

**Table S5 –Differentially Expressed (DE) genes shared by CycA dsRNA iECs, Myb dsRNA iECs and salivary gland devECs**

**Table S6 – Meta-analysis of RNA-Seq data for E2F1 regulated genes (from Dimova et al. 2003)(3).**

**Table S7 – Results of RNAi wing screen**

Stock# – Bloomington Drosophila Stock Center (BDSC) Stock number
Light blue – positive hit in the wing screen. Reduced size of L3-L4 region, increased bristle size.
Orange – Lethal. No adult flies after expression of the indicated dsRNA.

**Table S8 – Full list of fly strains and primers used**

Stock# – Bloomington Drosophila Stock Center (BDSC) Stock number

**Supplemental Figure Legends
Figure S1. Knockdown of CycB does not induce endoreplication.** S2 cells were treated with CycB dsRNA. **(A)** qRT-PCR quantification of CycB transcript in CycB dsRNA versus GFP dsRNA control cells. **(B)** Quantification of flow cytometry data for ploidy classes in GFP dsRNA and CycB dsRNA cells (mean and S.D. for N=2).

**Figure S2. Knockdown of CycA or Myb inhibits cell proliferation.** 500,000 cells were plated and treated with the indicated dsRNAs. The cells were counted once every 24h for 7 days (mean and S.D. for N=3).

**Figure S3. Statistical analysis of DE gene overlap between populations of endoreplicating cells.** Permutation testing was used to calculate *p*-values and fold enrichment of the pairwise overlap between CycA dsRNA iECs and Myb dsRNA iECs **(A)**, and three-way overlap among CycA dsRNA iECs, Myb dsRNA iECs, and Salivary Gland devECs **(B),** relative to chance (4). The graph shows the fold difference between the observed overlaps and those predicted by 100,000 iterations of random sampling values based on DE gene numbers. The vertical bar represents the median, and the extent of the boxes are the 5% and 95% quantiles (p< 1 x 10^-5^ for all comparisons).

**Figure S4. CycA dsRNA iECs and Myb dsRNA iECs have increased expression of genes involved in development.** Biological Process (BP) Gene Ontology (GO) category analysis was performed on genes that were upregulated at least Log2FC 0.5, with an FDR corrected q <0.05 in both the CycA dsRNA, and Myb dsRNA iECs relative to GFP dsRNA treated cells. The graph shows number of genes in the top 20 GO categories that were significantly enriched in both iEC types with color coding indicating FDR corrected q value for that class.

**Figure S5. CycA dsRNA iECs and Myb dsRNA iECs have decreased expression of genes required for mitosis.** BP GO category analysis was performed on genes that were downregulated at least Log2FC -0.5, with an FDR corrected q <0.05 in both the CycA dsRNA, and Myb dsRNA iECs relative to GFP dsRNA treated cells. The graph shows number of genes in the top 20 GO categories that were significantly enriched in both iEC types with color coding indicating FDR corrected q value for that class.

**Figure S6. iECs and devECs have decreased expression of Myb-induced genes that are required for mitosis.** Comparison of RNA-Seq results for iEC in culture and devEC in salivary glands. BP GO category analysis was performed on genes that were downregulated at least Log2FC -0.5, with an FDR of <0.05 in the CycA dsRNA, and Myb dsRNA iECs relative to GFP dsRNA treated cells and the salivary gland endocycling vs Brain-disc tissues. The graph shows the top 20 BP GO categories that were significantly enriched in the overlap of CycA dsRNA iECs, Myb dsRNA iECs , and salivary gland devECs.

**Figure S7. Knockdown of individual members of the CycA-Myb-aurB network is sufficient to induce endoreplication in wing imaginal discs.** Wing imaginal discs corresponding to dpp-GAL4 / UAS-dsRNA wing screen genotypes indicated in Figure 5. Red outlines indicate the border of the mRFP expression that corresponds to dpp-GAL4 expression. **(A)** A control wild type (w.t.) wing disc from a *dpp-GAL4, UAS-mRFP*; *UAS-GFP* animal. **(B)** A wing disc from a *dpp-GAL4, UAS-mRFP; UAS-CycA^dsRNA^* animal. Note the larger nuclei within the red border compared to cells outside. **(C-I)** Wing discs after expression of *UAS-Myb^dsRNA^* **(C)**, *AurB^dsRNA-1^* **(D),** *AurB^dsRNA-2^* **(E)**, *Incenp^dsRNA^* **(F)**, *Spc25^dsRNA^***(G),** *tum^dsRNA^* **(H),** or *pav^dsRNA^* **(I).**  Scale bars are 20μM.

**Figure S8. RT-qPCR quantification of RNAi knockdown in larval discs.** RT-qPCR quantification of the indicated transcripts in imaginal discs from different UAS-dsRNA strains normalized to that in wild type control discs. Each value on the X axis indicates both the dsRNA strain and the transcript measured after induction with a heat inducible GAL (N=2).

**Figure S9. Knockdown of *aurB* induces endoreplication whereas knockdown of *polo* induces a mitotic arrest in S2 cells. (A)** Flow cytometry of DNA content in propidium iodide labeled S2 cells 96 hours after treatment with either *GFP* dsRNA (control), *aurB* dsRNA or *polo* dsRNA. **(B)** Quantification of EdU and pH3 labeling in cells after treatment with the indicated dsRNAs (mean and S.E.M. for N=3, * - p < 0.05, ** p < 0.01, ns – not significant).

**Figure S10. Myb over-expression does not inhibit endoreplication after CycA knockdown.**

Induction of endoreplication by knockdown of CycA is not suppressed by overexpressing Myb. Quantification of nuclear area of ovary follicle cells in stage 6 egg chambers after heat inducing the following genotypes: 1) *UAS-GFP/+; Hsp70-GAL4, UAS-mRFP/+*, 2) *UAS-GFP/+; Hsp70-GAL4, UAS-mRFP-Myb /+*, 3) *(UAS-CycA dsRNA/+; Hsp70-GAL4, UAS-mRFP / +*, and 4)

*UAS-CycA dsRNA/+; Hsp70-GAL4, UAS-mRFP-Myb / +*. Each dot represents the nuclear area of a single cell divided by the mean area of controls (machine units). Mean and S.D. N>5 egg chambers, n>100 cells, ns – not significant).

**References**

1. Benjamini Y, Hochberg Y. Controlling the False Discovery Rate: A Practical and Powerful Approach to Multiple Testing. Journal of the Royal Statistical Society Series B (Methodological). 1995;57(1):289-300.

2. Hu Y, Flockhart I, Vinayagam A, Bergwitz C, Berger B, Perrimon N, et al. An integrative approach to ortholog prediction for disease-focused and other functional studies. BMC Bioinformatics. 2011;12(1):357.

3. Dimova DK, Stevaux O, Frolov MV, Dyson NJ. Cell cycle-dependent and cell cycle-independent control of transcription by the Drosophila E2F/RB pathway. Genes Dev. 2003;17(18):2308-20.

4. Phipson B, Smyth GK. Permutation P-values should never be zero: calculating exact P-values when permutations are randomly drawn. Stat Appl Genet Mol Biol. 2010;9:Article39.
