# Supplementary figures and images for: A Cyclin A—Myb-MuvB—Aurora B network regulates the choice between mitotic cycles and polyploid endoreplication cycles
Source: PLoS Genet. 2019 Jul 10;15(7):e1008253. doi: 10.1371/journal.pgen.1008253 (PMC6645565; doi:10.1371/journal.pgen.1008253)

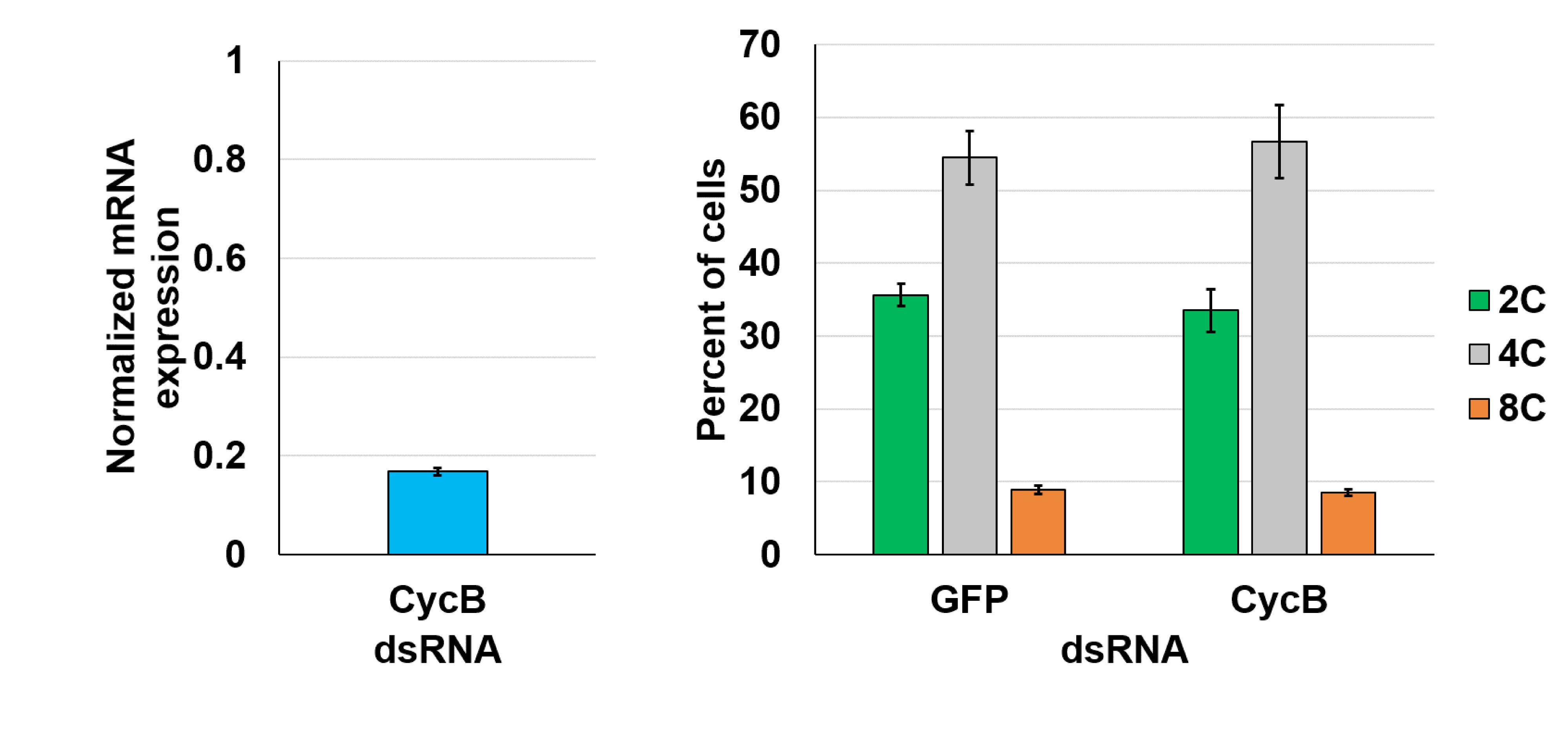

Supplement: S1 Fig — S2 cells were treated with CycB dsRNA. (A) qRT-PCR quantification of CycB transcript in CycB dsRNA versus GFP dsRNA control cells. (B) Quantification of flow cytometry data for ploidy classes in GFP dsRNA and CycB dsRNA cells (mean and S.D. for N = 2). (TIF) [file pgen.1008253.s010.tif]

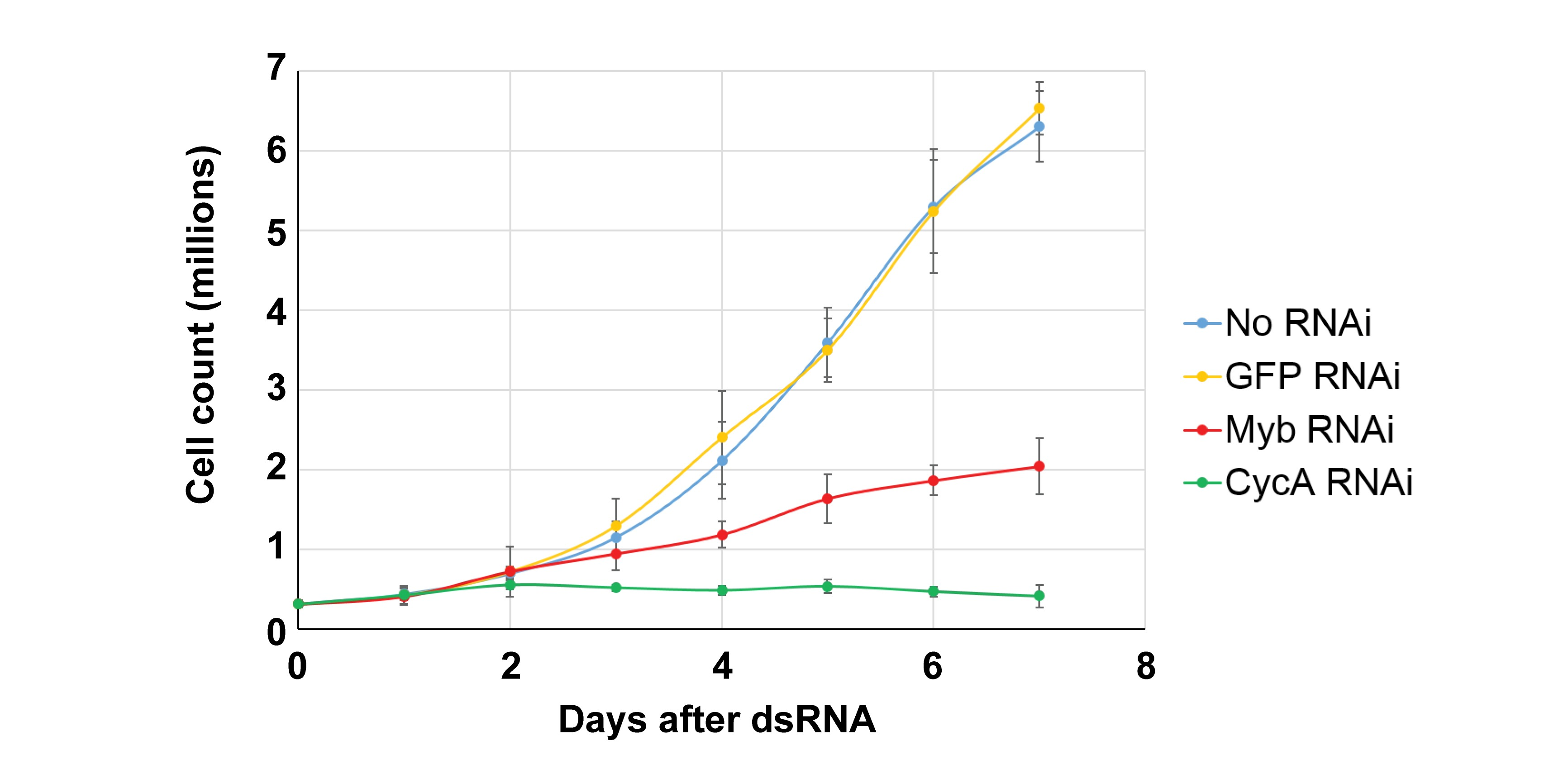

Supplement: S2 Fig — 500,000 cells were plated and treated with the indicated dsRNAs. The cells were counted once every 24h for 7 days (mean and S.D. for N = 3). (TIF) [file pgen.1008253.s011.tif]

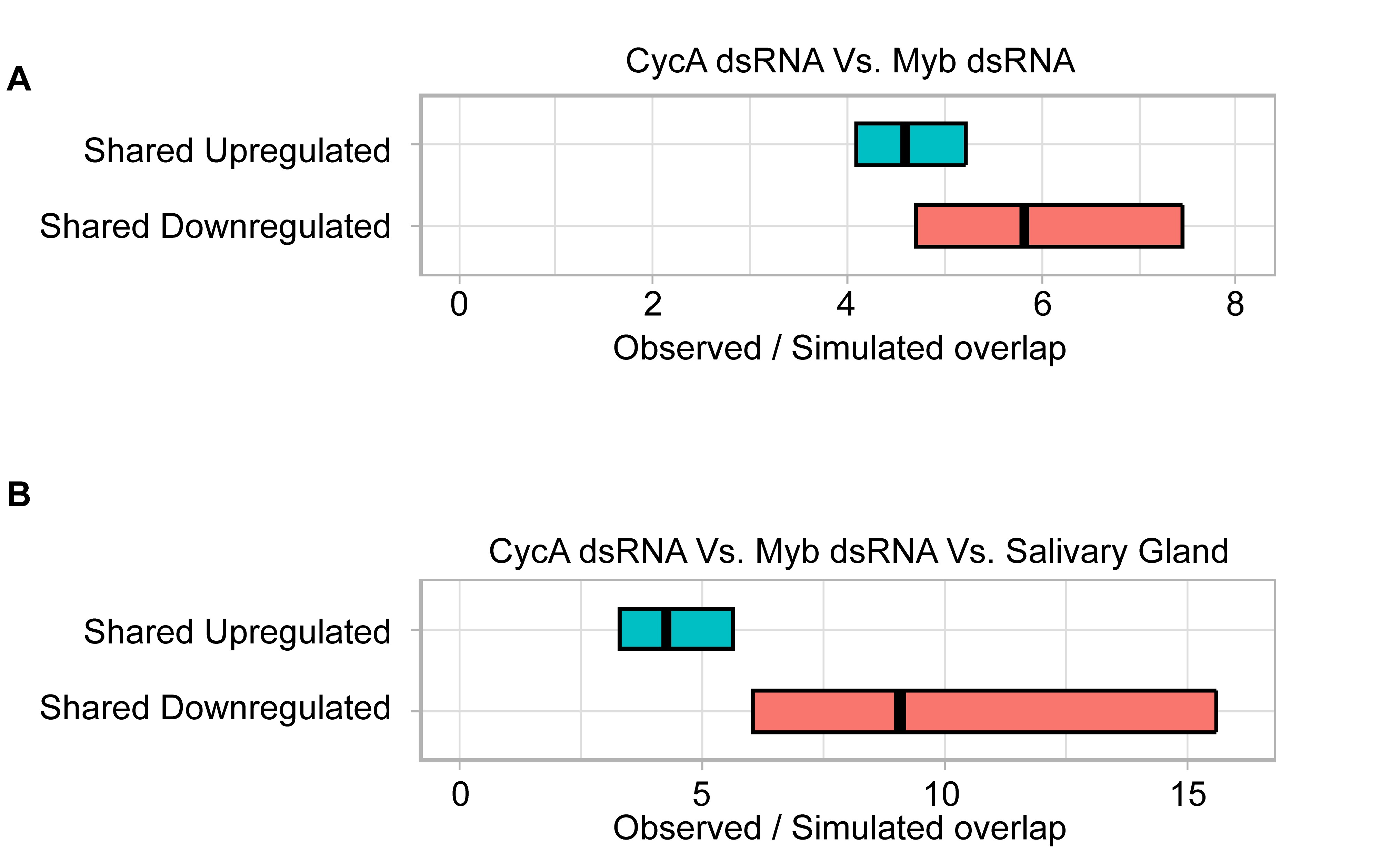

Supplement: S3 Fig — Permutation testing was used to calculate p-values and fold enrichment of the pairwise overlap between CycA dsRNA iECs and Myb dsRNA iECs (A), and three-way overlap among CycA dsRNA iECs, Myb dsRNA iECs, and Salivary Gland devECs (B), relative to chance (4). The graph shows the fold difference between the observed overlaps and those predicted by 100,000 iterations of random sampling values based on DE gene numbers. The vertical bar represents the median, and the extent of the boxes are the 5% and 95% quantiles (p< 1 x 10^−5^ for all comparisons). (TIF) [file pgen.1008253.s012.tif]

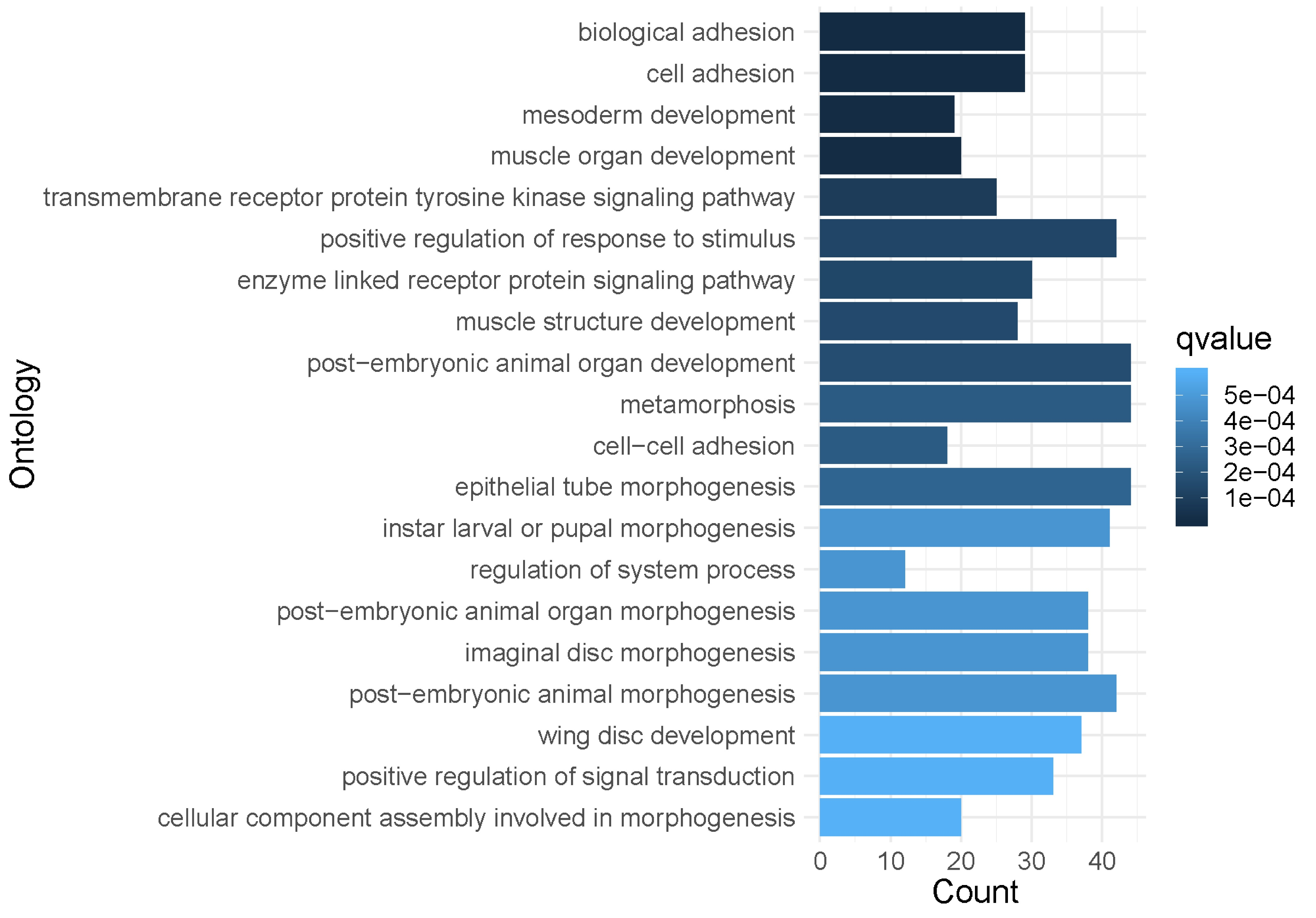

Supplement: S4 Fig — Biological Process (BP) Gene Ontology (GO) category analysis was performed on genes that were upregulated at least Log2FC 0.5, with an FDR corrected q <0.05 in both the CycA dsRNA, and Myb dsRNA iECs relative to GFP dsRNA treated cells. The graph shows number of genes in the top 20 GO categories that were significantly enriched in both iEC types with color coding indicating FDR corrected q value for that class. (TIF) [file pgen.1008253.s013.tif]

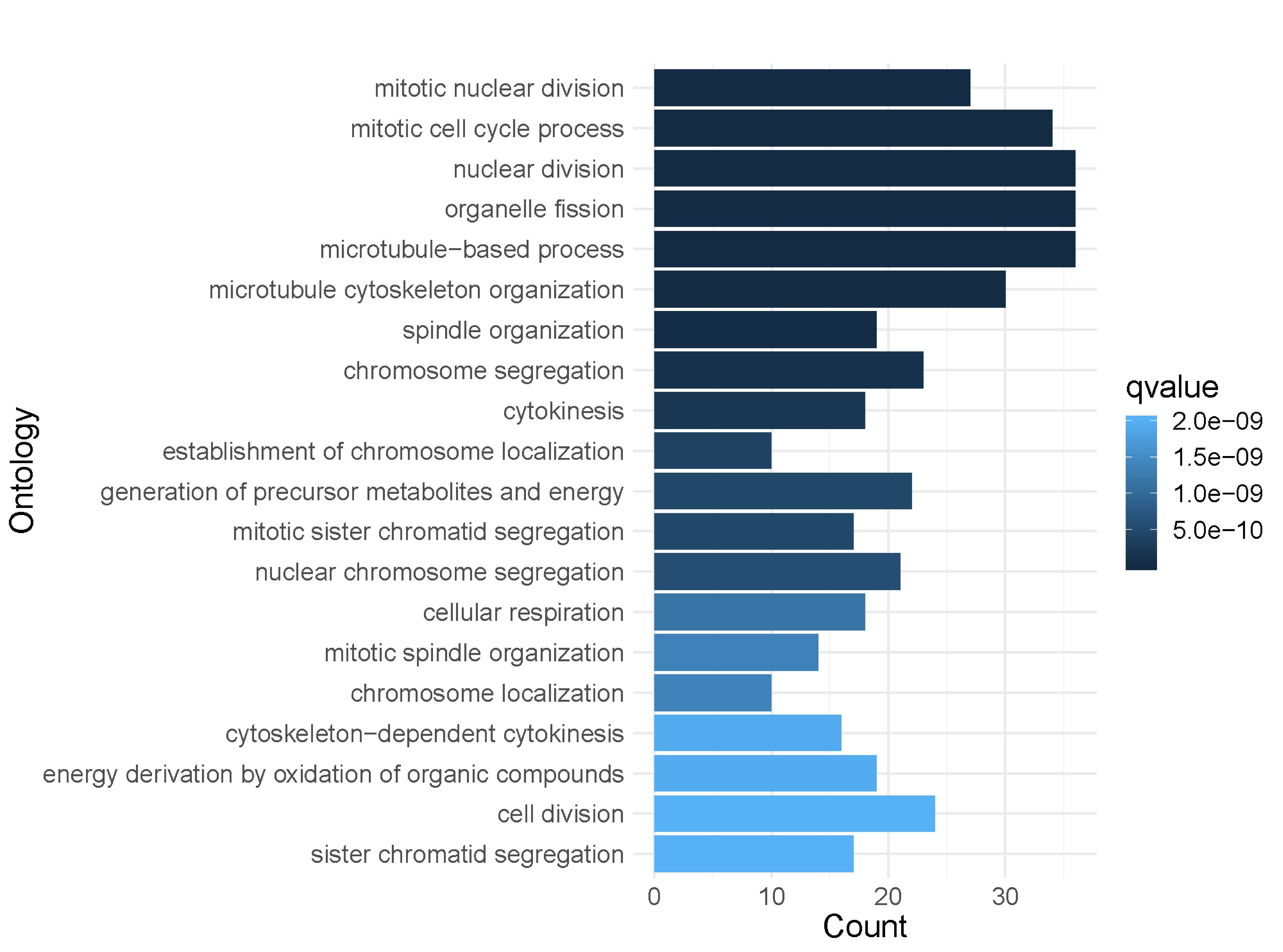

Supplement: S5 Fig — BP GO category analysis was performed on genes that were downregulated at least Log2FC -0.5, with an FDR corrected q <0.05 in both the CycA dsRNA, and Myb dsRNA iECs relative to GFP dsRNA treated cells. The graph shows number of genes in the top 20 GO categories that were significantly enriched in both iEC types with color coding indicating FDR corrected q value for that class. (TIF) [file pgen.1008253.s014.tif]

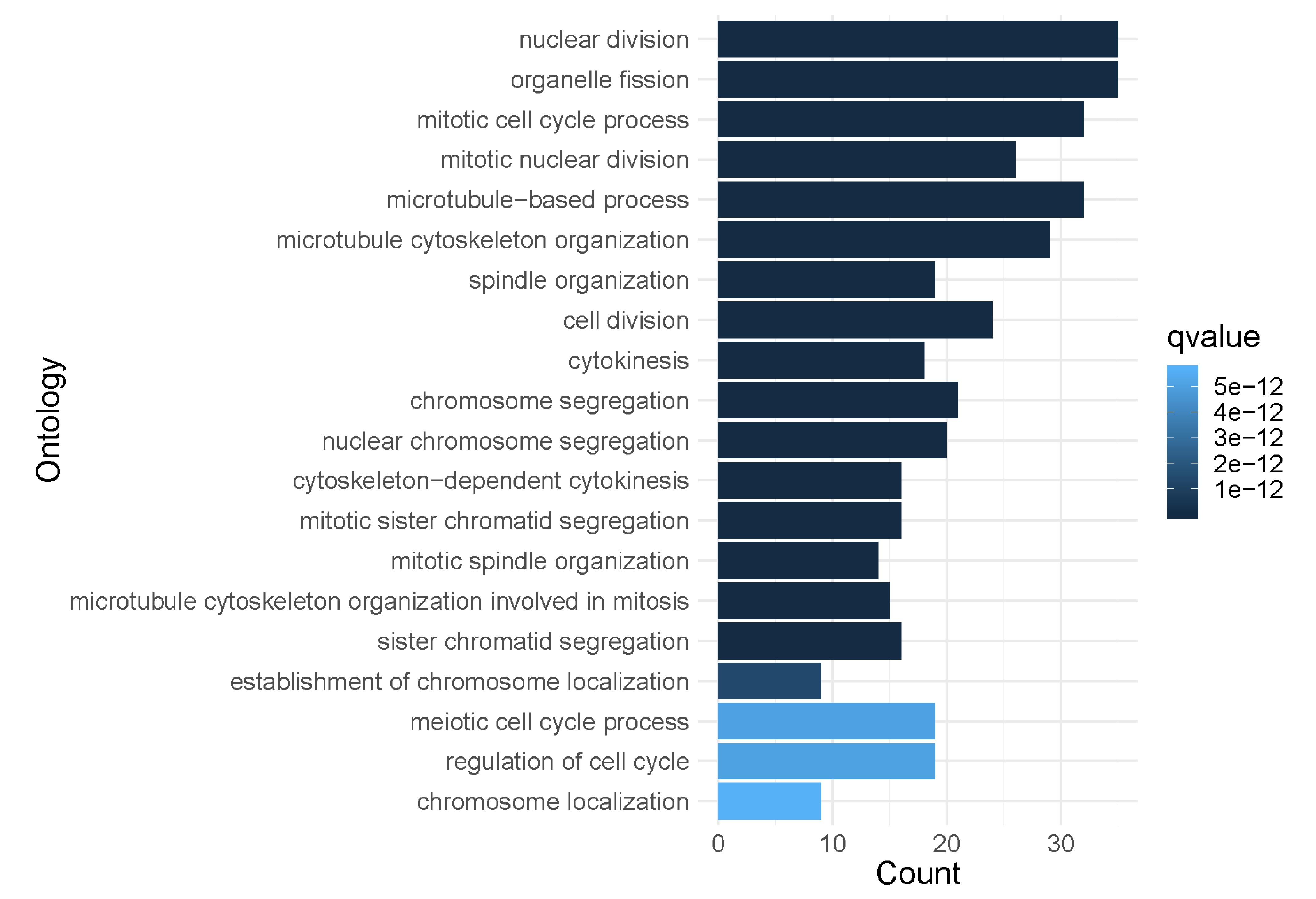

Supplement: S6 Fig — Comparison of RNA-Seq results for iEC in culture and devEC in salivary glands. BP GO category analysis was performed on genes that were downregulated at least Log2FC -0.5, with an FDR of <0.05 in the CycA dsRNA, and Myb dsRNA iECs relative to GFP dsRNA treated cells and the salivary gland endocycling vs Brain-disc tissues. The graph shows the top 20 BP GO categories that were significantly enriched in the overlap of CycA dsRNA iECs, Myb dsRNA iECs, and salivary gland devECs. (TIF) [file pgen.1008253.s015.tif]

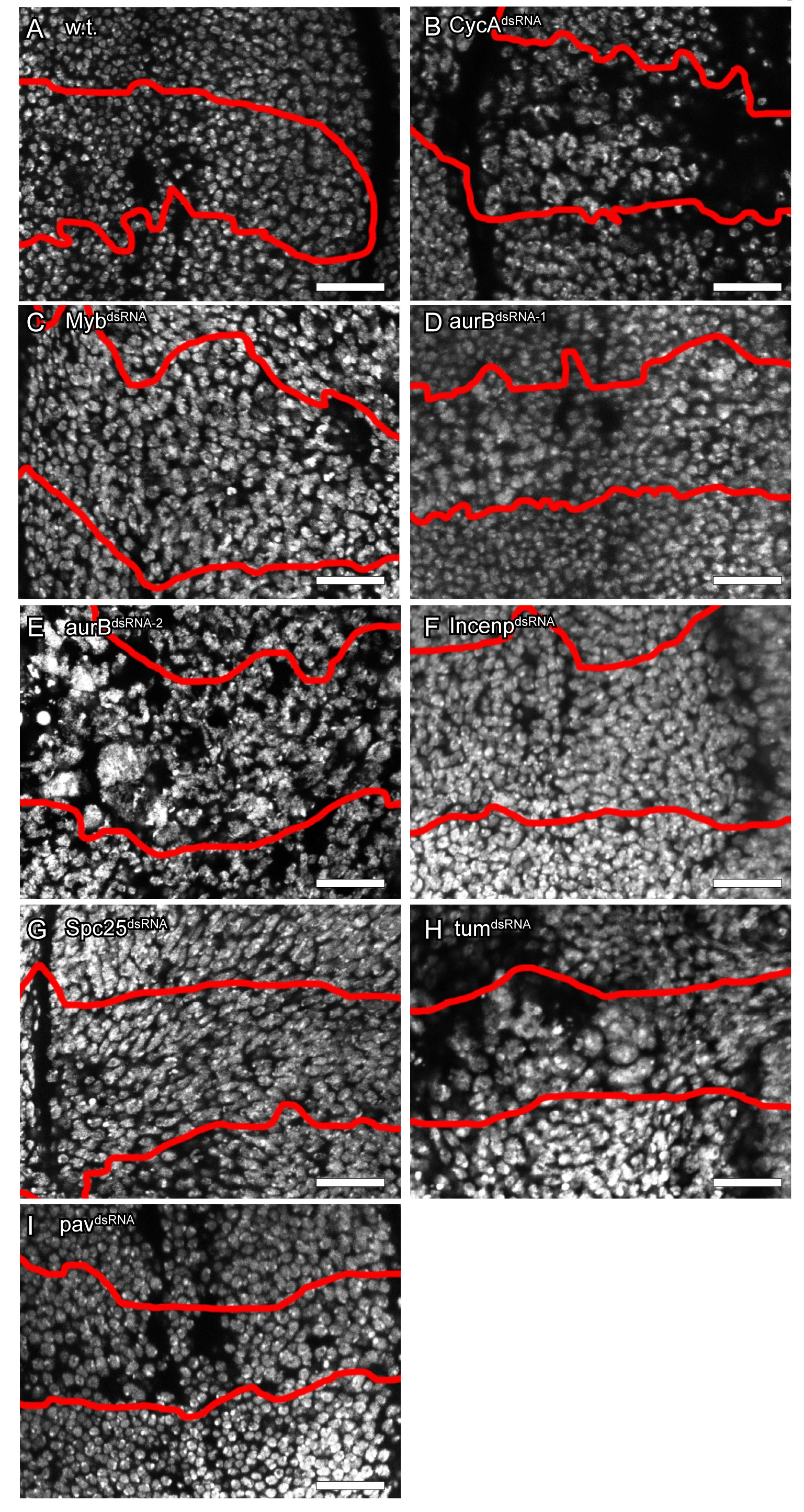

Supplement: S7 Fig — Wing imaginal discs corresponding to dpp-GAL4 / UAS-dsRNA wing screen genotypes indicated in Fig 5. Red outlines indicate the border of the mRFP expression that corresponds to dpp-GAL4 expression. (A) A control wild type (w.t.) wing disc from a dpp-GAL4, UAS-mRFP; UAS-GFP animal. (B) A wing disc from a dpp-GAL4, UAS-mRFP; UAS-CycAdsRNA animal. Note the larger nuclei within the red border compared to cells outside. (C-I) Wing discs after expression of UAS-MybdsRNA (C), AurBdsRNA-1 (D), AurBdsRNA-2 (E), IncenpdsRNA (F), Spc25dsRNA(G), tumdsRNA (H), or pavdsRNA (I). Scale bars are 20μM. (TIF) [file pgen.1008253.s016.tif]

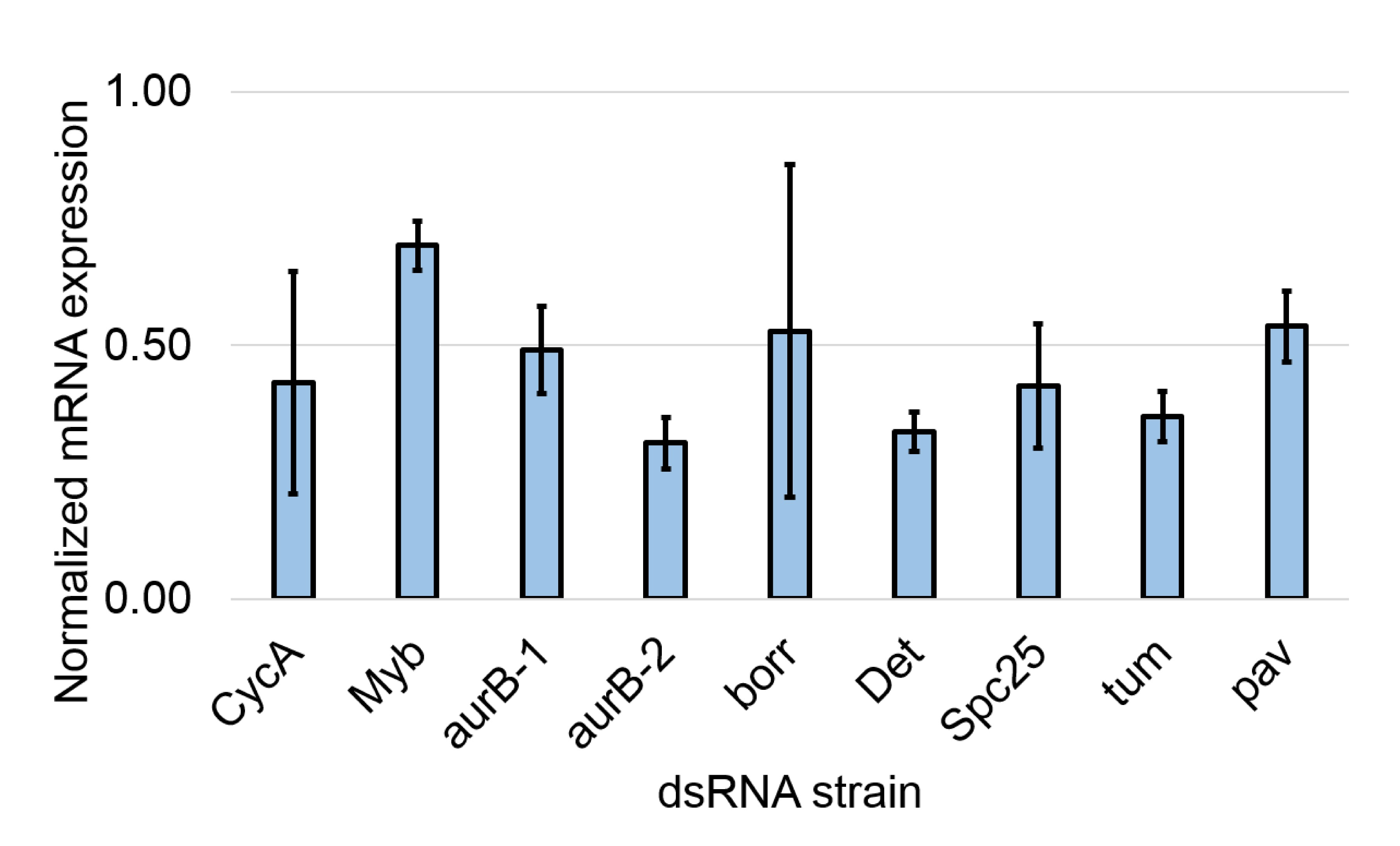

Supplement: S8 Fig — RT-qPCR quantification of the indicated transcripts in imaginal discs from different UAS-dsRNA strains normalized to that in wild type control discs. Each value on the X axis indicates both the dsRNA strain and the transcript measured after induction with a heat inducible GAL (N = 2). (TIF) [file pgen.1008253.s017.tif]

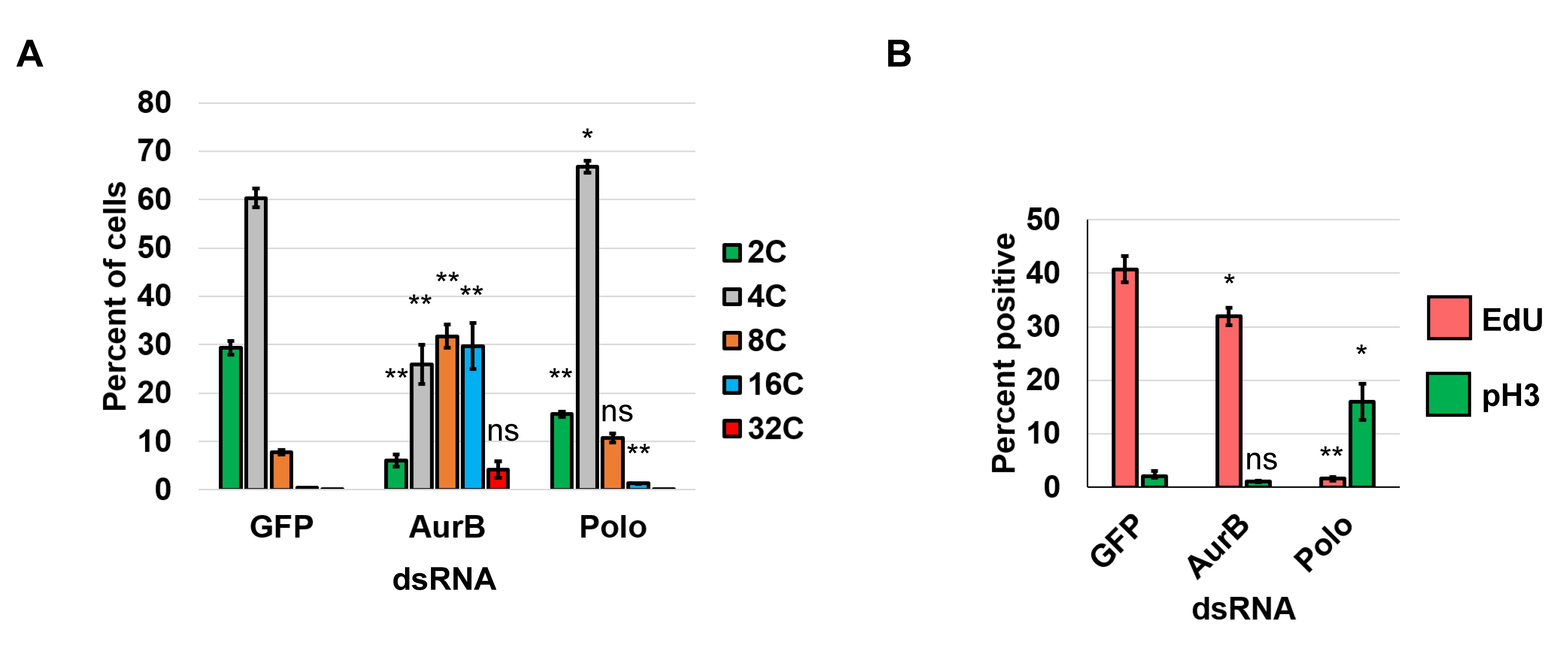

Supplement: S9 Fig — (A) Flow cytometry of DNA content in propidium iodide labeled S2 cells 96 hours after treatment with either GFP dsRNA (control), aurB dsRNA or polo dsRNA. (B) Quantification of EdU and pH3 labeling in cells after treatment with the indicated dsRNAs (mean and S.E.M. for N = 3, *—p < 0.05, ** p < 0.01, ns—not significant). (TIF) [file pgen.1008253.s018.tif]

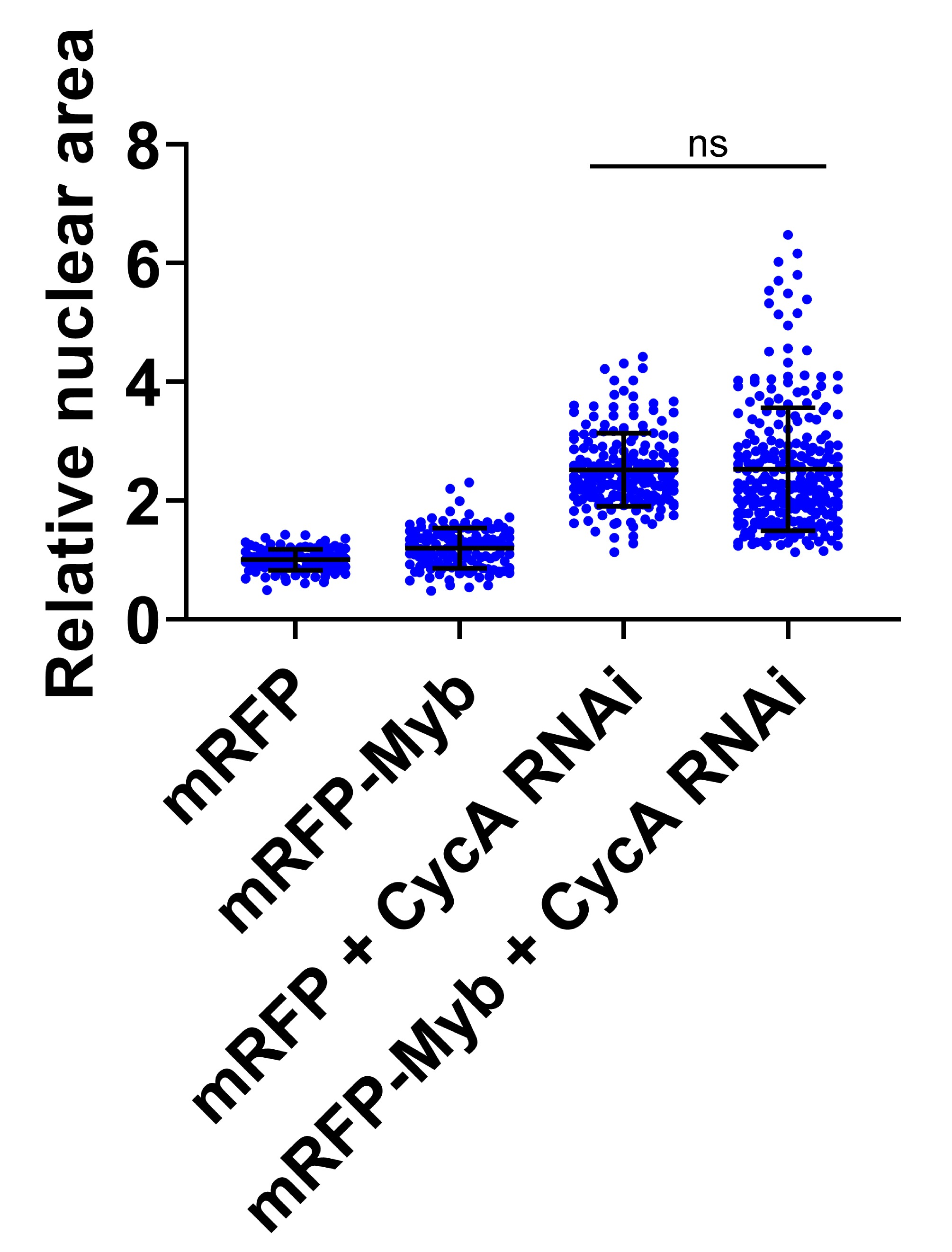

Supplement: S10 Fig — Induction of endoreplication by knockdown of CycA is not suppressed by overexpressing Myb. Quantification of nuclear area of ovary follicle cells in stage 6 egg chambers after heat inducing the following genotypes: 1) UAS-GFP/+; Hsp70-GAL4, UAS-mRFP/+, 2) UAS-GFP/+; Hsp70-GAL4, UAS-mRFP-Myb /+, 3) (UAS-CycA dsRNA/+; Hsp70-GAL4, UAS-mRFP / +, and 4) UAS-CycA dsRNA/+; Hsp70-GAL4, UAS-mRFP-Myb / +. Each dot represents the nuclear area of a single cell divided by the mean area of controls (machine units). Mean and S.D. N≥5 egg chambers, n≥100 cells, ns—not significant). (TIF) [file pgen.1008253.s019.tif]
